# Bioactive Polyketides and Benzene Derivatives from Two Mangrove Sediment-Derived Fungi in the Beibu Gulf

**DOI:** 10.3390/md21060327

**Published:** 2023-05-26

**Authors:** Bo Peng, Jian Cai, Zimin Xiao, Manli Liu, Xinlong Li, Bin Yang, Wei Fang, Yi-You Huang, Chunmei Chen, Xuefeng Zhou, Huaming Tao

**Affiliations:** 1 Institute for Environmental and Climate Research, Jinan University, Guangzhou 511443, China; pengbo@jnu.edu.cn; 2 CAS Key Laboratory of Tropical Marine Bio-Resources and Ecology, Guangdong Key Laboratory of Marine Materia Medica, South China Sea Institute of Oceanology, Chinese Academy of Sciences, Guangzhou 510301, China; caijian19@mails.ucas.ac.cn (J.C.); 13409379936@163.com (X.L.); yangbin@scsio.ac.cn (B.Y.); 3 Guangdong Provincial Key Laboratory of Chinese Medicine Pharmaceutics, School of Traditional Chinese Medicine, Southern Medical University, Guangzhou 510515, China; 15917491112@163.com; 4 Hubei Biopesticide Engineering Research Center, Hubei Academy of Agricultural Science, Wuhan 430064, China; manli.liu@nberc.com (M.L.); wei.fang@nberc.com (W.F.); 5 Key Laboratory of Tropical Biological Resources of Ministry of Education, School of Pharmaceutical Sciences, Hainan University, Haikou 570228, China; hyyou@hainanu.edu.cn

**Keywords:** mangrove sediment-derived fungi, polyketide, stachylines, biological activity

## Abstract

To discover bioactive natural products from mangrove sediment-derived microbes, a chemical investigation of the two Beibu Gulf-derived fungi strains, *Talaromyces* sp. SCSIO 41050 and *Penicillium* sp. SCSIO 41411, led to the isolation of 23 natural products. Five of them were identified as new ones, including two polyketide derivatives with unusual acid anhydride moieties named cordyanhydride A ethyl ester (**1**) and maleicanhydridane (**4**), and three hydroxyphenylacetic acid derivatives named stachylines H–J (**10**–**12**). Their structures were determined by detailed nuclear magnetic resonance (NMR) and mass spectroscopic (MS) analyses, while the absolute configurations were established by theoretical electronic circular dichroism (ECD) calculation. A variety of bioactive screens revealed three polyketide derivatives (**1**–**3**) with obvious antifungal activities, and **4** displayed moderate cytotoxicity against cell lines A549 and WPMY-1. Compounds **1** and **6** at 10 μM exhibited obvious inhibition against phosphodiesterase 4 (PDE4) with inhibitory ratios of 49.7% and 39.6%, respectively, while **5**, **10**, and **11** showed the potential of inhibiting acetylcholinesterase (AChE) by an enzyme activity test, as well as in silico docking analysis.

## 1. Introduction

The mangrove wetland ecosystems, located at tropical and subtropical intertidal estuarine zones, possess rich biodiversity and include an enormous diversity of microorganisms [1]. From these, a vast range of fungi species in mangrove sediment play a vital role in biogeochemical cycles to sustain the mangrove wetland ecosystems [2]. Mangrove sediment-derived fungi are widely considered to be a pivotal and prolific reservoir of structurally unique and biologically active secondary metabolites with promising medicinal, agricultural, or industrial applications [2,3].

The genus *Talaromyces* is widely distributed in marine environments, soil, plants, and foods. The extreme living conditions have led the fungi to develop more specific metabolic patterns, and marine-derived *Talaromyces* spp. can produce a number of structurally diverse substances with a wide range of bioactivities, such as anti-inflammatory meroterpenoids, thioester containing benzoate derivatives that exhibit α-glucosidase inhibitory activity, and oxaphenalenone dimers with broad antibacterial activity [4].

*Penicillium* species are among the most widespread fungal organisms on earth and contains more than 350 species. Many *Penicillium* species can produce plentiful secondary metabolites, such as alkaloids [5], polyketides [6] and terpenoids [7], that can ascribe specific structural characteristics and significant biological activities.

**Figure 1:**
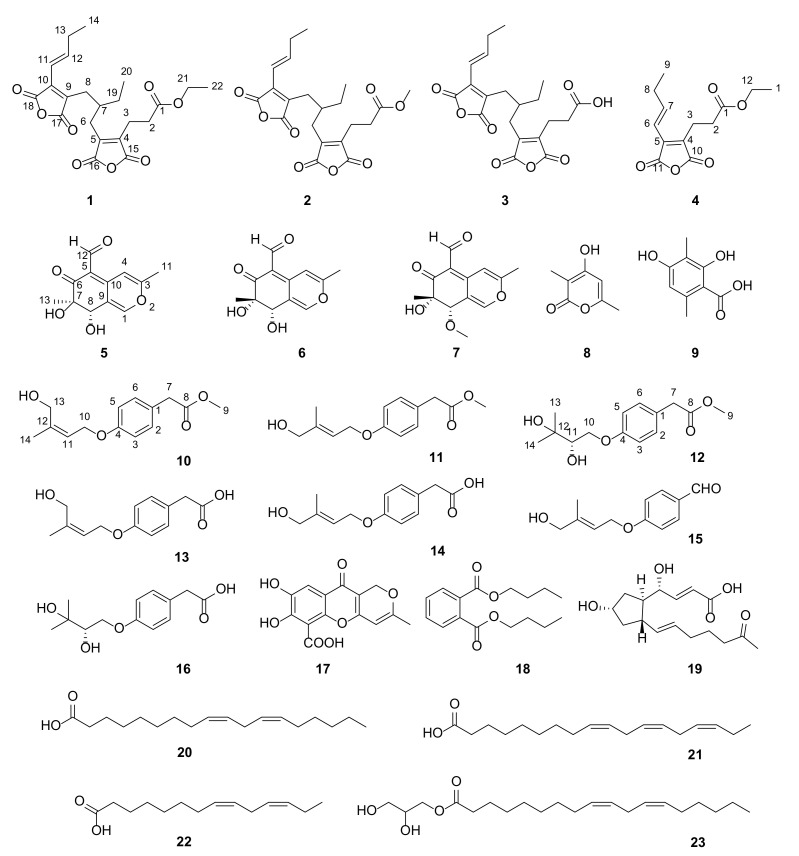
Structures of compounds **1**–**23**.

As part of our research on discovering structurally novel and bioactive natural products from mangrove sediment-derived fungi [5,8,9], two fungi strains of *Talaromyces* sp. SCSIO 41050 and *Penicillium* sp. SCSIO 41411, isolated from a mangrove sediment sample, collected from Gaoqiao mangrove wetland in the Zhanjiang coastline of the northern part of Beibu Gulf, attracted our attention for the characterization of their HPLC-DAD profiles. Further chemical investigations of their crude extract led to the isolation of 23 natural products, including four maleic anhydride polyketides (**1**–**4**), three austdiol polyketides derivates (**5**–**7**), a 2*H*-pyran-2-one derivate (**8**), a benzoic acid derivate (**9**), seven stachyline derivates (**10**–**16**), and seven carboxylic acids and ester derivatives (**17**–**23**) (Figure 1). Five of them were identified as new ones: two polyketides named cordyanhydride A ethyl ester (**1**) and maleicanhydridane (**4**) from *Talaromyces* sp. SCSIO 41050, and three benzene derivatives called stachylines H–J (**10**–**12**) from *Penicillium* sp. SCSIO 41411. Herein, details of the isolation, structure elucidation, and biological activities of these compounds are described.

## 2. Results and Discussion

### 2.1. Structural Determination

**Figure 2:**
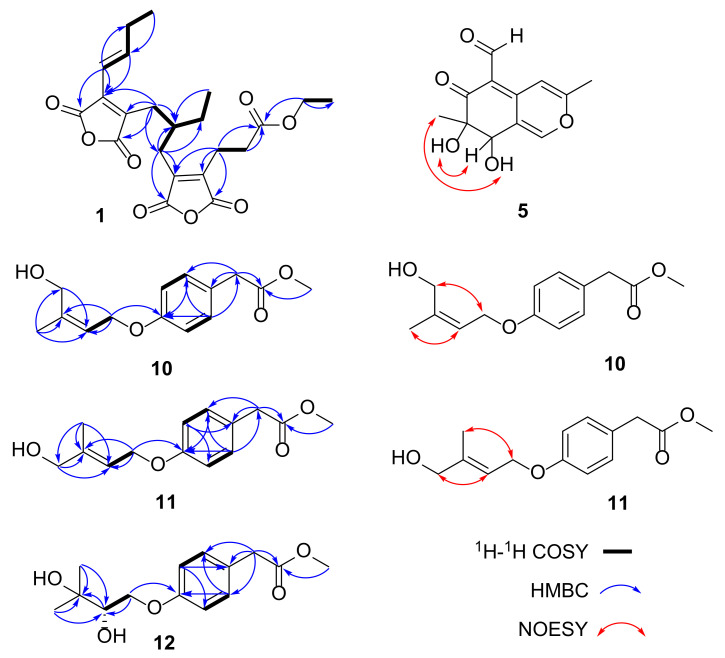
Key ^1^H-^1^H COSY, HMBC, and NOESY correlations of **1**, **5**, and **10**–**12**.

Compound **1** was obtained as a yellow oil, and its molecular formula was established as C_22_H_26_O_8_ by HRESIMS ion peak at *m*/*z* 419.1701 [M + H]^+^ (calcd for C_22_H_27_O_8_^+^, 419.1700). A detailed analysis of ^1^H NMR data (Table 1) of **1** exhibited the presence of two olefinic protons at *δ*_H_ 7.08 (dt, *J* = 15.5, 6.5 Hz, H-12) and 6.43 (dt, *J* = 15.5, 1.5 Hz, H-11); one methine at *δ*_H_ 2.05 (m, H-7); seven methylenes (including one oxygenated) at *δ*_H_ 4.05 (q, *J* = 7.0 Hz, H_2_-21), 2.68 (t, *J* = 8.0 Hz, H_2_-3), 2.58 (dd, *J* = 9.5, 7.0 Hz, H_2_-2), 2.47/2.40 (H_2_-6), 2.47/2.05 (H_2_-7), 2.28 (m, H_2_-13), and 1.30 (m, H_2_-19); and three methyls at *δ*_H_ 1.17 (t, *J* = 7.0 Hz, H_3_-19), 1.05 (t, *J* = 7.5 Hz, H_3_-14), and (d, *J* = 0.88 Hz, H_3_-20). The ^13^C NMR data and HSQC spectrum displayed 22 carbon signals including five ester carbons at *δ*_C_ 171.5 (C-1), 166.2 (C-17), 166.0 (C-16), 165.5 (C-15), and 164.5 (C-18); four olefinic tertiary carbons at *δ*_C_ 144.1 (C-4), 143.0 (C-5), 138.1 (C-9), and 137.7 (C-10); two olefinic methine carbons at *δ*_C_ 147.7 (C-12) and 117.0 (C-11); one methine carbon at *δ*_C_ 37.6 (C-7); seven methylene carbons (including one oxygenated) at *δ*_C_ 60.3 (C-21), 30.8 (C-2), 27.9 (C-8), 27.4 (C-6), 26.6 (C-13), 25.7 (C-19), and 19.4 (C-3); and three methyl carbons at *δ*_C_ 14.0 (C-22), 12.5 (C-14), and 10.7 (C-20). The ^1^H-^1^H COSY correlations (Figure 2) of H_2_-2/H_2_-3, H_2_-6/H-7/H_2_-8, H-7/H_2_-19/H_3_-20, and H-11/H-12/H_2_-13/H_3_-14 revealed partial structure of CH_2_-2/CH_2_-3, CH_2_-6/CH-7(CH_2_-19/CH_3_-20)/CH_2_-8, and CH-11/CH-12/CH_2_-13/CH_3_-14. The above NMR data indicated that the structural skeleton of **1** was similar to that of the co-isolated cordyanhydride A methyl ester (**2**) [10], with two acid anhydride moieties. The main distinction was the presence of an ethyl ester group at C-1 of **1** instead of the methyl ester group in **2**, which was supported by key ^1^H-^1^H COSY correlation of H_2_-21/H_3_-22 and HMBC correlations (Figure 2) from H_2_-21 to C-1 and C-22. Thus, the planar structure of **1** was defined as shown in Figure 1, and the other HMBC correlations supported the deduction. The configurations of the Δ^11^ double bonds were all deduced as *E* based on the large coupling constant *J*_H-11/H-12_ = 15.5 Hz. Due to the weak Cotton effect and the failed single crystal cultivation experiment, the absolute configuration of C-7 was unsolved. Finally, compound **1** was identified to be a new maleic anhydride derivative named cordyanhydride A ethyl ester (**1**).

Compound **4** was isolated as a yellow oil. Its molecular formula was established as C_13_H_17_O_5_ by HRESIMS ion peak at *m*/*z* 253.1073 [M + H]^+^ (calculated for C_13_H_17_O_5_^+^, 253.1071). Comparison of NMR spectroscopic data of **4** (Table 1) with **1** revealed that **4** shared part of the structure of **1**, with one acid anhydride moiety. By further analysis of the chemical shift, the coupling constant and the molecular formula, **4** was determined as shown in Figure 1. The structure had been reported as a synthetic product in a patent without NMR data and trivial name [11]. Herein, it was discovered as a new natural product and named maleicanhydridane (**4**).

Compounds bearing acid anhydride moieties are rare in nature. Cordyanhydrides A and B, bearing two and three acid anhydride moieties, were originally described from the insect pathogen fungus *Cordyceps pseudomilitaris* [12] and Amazonian endophytic *Talaromyces* fungi [10], with the absolute configuration unsolved. To the best of our knowledge, this study is the first example to obtain cordyanhydride derivatives from marine-derived microbes. In addition, **4** should be a precursor compound for the biosynthesis of **1**–**3**.

**Figure 3:**
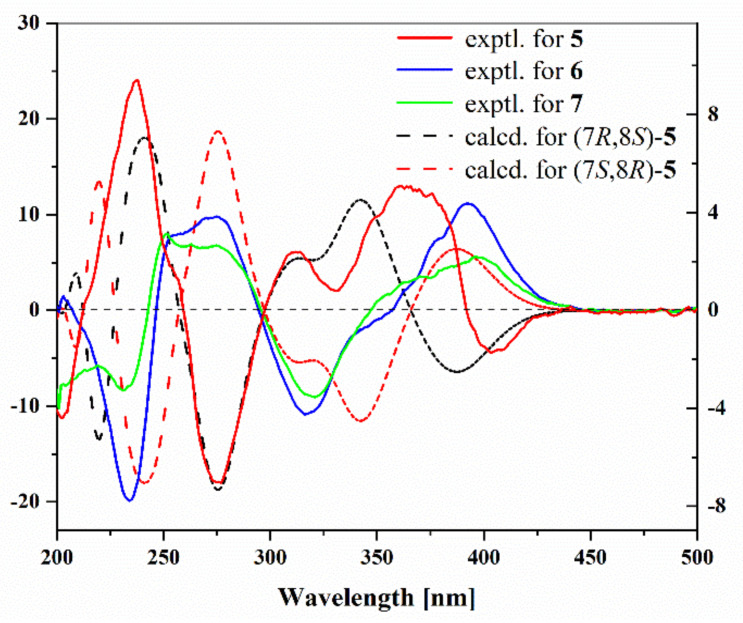
Experimental ECD spectra of **5**–**7** and calculational ECD spectrum of **5**.

Compound **5** has been reported as a fungal metabolite [13] and was isolated as an epimer at C-7 of 7-epiaustdiol (**6**) [14] with almost identical NMR data. The absolute configurations of 7-epiaustdiol (**6**) and 8-*O*-methylepiaustdiol (**7**) were determined as shown in Figure 1, because they share the matched experimental ECD curve (Figure 3) and have similar OR values (${\left[\alpha \right]}_{D}^{25}$ +172 (*c* 0.05, CH_3_OH) and ${\left[\alpha \right]}_{D}^{25}$ +160 (*c* 0.05, CH_3_OH)), similar to reports in the literature. In order to determine the absolute configurations of C-7/C-8 of **5**, the NOESY analysis and ECD calculation methods were used. The NOESY correlations of OH-7/H-8 and OH-8/H_3_-13 (Figure 2) supported the *trans* configuration of OH-7 and OH-8. The Boltzmann-weighted ECD curves of 7*R*,8*S*-**5** and 7*S*,8*R*-**5** were calculated and compared with the experimental ECD curve (Tables S1 and S2), which led to the determination of the 7*R*,8*S* absolute configuration of **5** (Figure 3). Thus, **5** was assigned as (7*R*, 8*S*)-austdiol (**5**).

Compound **10** was isolated as a brown oil, and its molecular formula was determined as C_14_H_18_O_4_ by HRESIMS ion peak at *m*/*z* 273.1097 [M + Na]^+^ (calculated for C_14_H_18_NaO_4_^+^, 273.1097), corresponding to six indices of hydrogen deficiency. The ^1^H NMR data (Table 2) of **10** displayed the presence of a characteristic for *para*-substituted aromatic ring [*δ*_H_ 7.15 (d, *J* = 8.5 Hz, H-2 and H-6) and 6.86 (d, *J* = 8.6 Hz, H-3 and H-5)], three methylene groups [*δ*_H_ 4.57 (d, *J* = 6.6 Hz, H_2_-10), 4.00 (s, H_2_-13), and 3.58 (s, H_2_-7)], a trisubstituted double bond [*δ*_H_ 5.43 (t, *J* = 6.3 Hz, H-11)], and two methyl groups [*δ*_H_ 3.59 (s, H_3_-9) and 1.75 (s, H_3_-14)]. The ^13^C NMR data (Table 2) of **10** displayed 14 carbon resonances consistent with four non-protonated carbons (including three sp^2^ carbons and a carbonyl), five sp^2^ methine groups, three methylene groups, and two methyl groups (one of them oxygenated). The ^1^H-^1^H COSY spectrum (Figure 2) indicated the presence of three independent spin systems of H-2/H-3, H-5/H-6, and H_2_-10/H-11. Comparison of its NMR data with those of stachyline G from *Mortierella* sp. revealed closed similarities except for the presence of an additional oxygenated methyl signal [15]. Extensive analysis of its HMBC spectrum revealed key signals from H_3_-9 to C-8, indicating that the methoxy was linked to C-8 and formed a methyl ester group. The geometric configuration of the Δ^10,11^ double bond was determined to be in *Z* configuration by the NOESY correlations (Figure 2) of H_2_-13 with H_2_-10, and H_3_-14 and H-11. The gross structure was constructed as shown in Figure 1 and named stachyline H (**10**).

Compound **11** was isolated as a brown oil, and its molecular formula was designated as C_14_H_18_O_4_ by HRESIMS ion peak at *m*/*z* 268.1540 [M + NH_4_]^+^ (calculated for C_14_H_22_NO_4_^+^, 268.1543). Comparison of NMR spectroscopic data of **11** with **10** indicated that they shared the same planar structures, supported by the HMBC and COSY correlations (Figure 2). Upon, detailed interpretation of its ^13^C NMR data, we found that the C-13 resonance was shielded from *δ*_C_ 21.0 in **10** to *δ*_C_ 13.8 in **11**, while the C-14 resonance was deshielded from *δ*_C_ 59.8 in **10** to *δ*_C_ 65.4 in **11**. The above obvious differences in the chemical shifts at C-13 and C-14 hinted that the geometric configuration of the Δ^10,11^ double bond is different in both compounds. The NOESY spectrum (Figure 2) of **11** revealed key signals of H_3_-14 with H_2_-10, and H_2_-13 and H-11, indicating the double bond Δ^10,11^ was in *E* configuration. Accordingly, it was elucidated and named stachyline I (**11**).

Compound **12** was obtained as a colorless oil, and its molecular formula of C_14_H_20_O_5_ was deduced from the positive HRESIMS ion peak at *m*/*z* 286.1651 [M + NH_4_]^+^ (calculated for C_14_H_24_NO_5_^+^, 286.1649), implying five degrees of hydrogen deficiency. The ^1^H NMR data (Table 2) of **12** displayed the series of typical proton signals responsive for a *para*-substituted aromatic ring [*δ*_H_ 7.15 (d, *J* = 8.5 Hz, H-2 and H-6) and 6.88 (d, *J* = 8.6 Hz, H-3 and H-5)], two methylene groups [*δ*_H_ 3.58 (s, H_2_-7), 4.19 (dd, *J* = 10.1, 2.3 Hz, H-10a), and 3.76 (dd, *J* = 10.0, 8.0 Hz, H-10b)], a oxygenated methine [*δ*_H_ 3.52 (d, *J* = 7.9 Hz, H-11)], and three methyl groups [*δ*_H_ 3.59 (s, H_3_-9), 1.13 (s, H_3_-13), and 1.07 (s, H_3_-14)]. The ^13^C NMR data (Table 2) of **12** displayed 14 carbon resonances consistent with four non-protonated carbons (including one sp^3^ carbon, two sp^2^ carbons and a carbonyl), five sp^2^ methine groups (including one oxygenated and four sp^2^ hybridized), two methylene groups (one of them oxygenated), and three methyl groups (one of them oxygenated). Analysis of its NMR data revealed that the structure of **12** closely resembled that of **10**. The difference was the replacement of signals for the 4-hydroxy-2-en-3-methylbutoxy unit substituted at the C-4 position in **10** with those for the 2,3-dihydroxy-3-methylbutoxy moiety in **12**. The ^1^H-^1^H COSY correlation of H_2_-10/H-11 and the HMBC correlations of H_2_-10 with C-4 and C-11, H-11 with C-12, and H_3_-13/H_3_-14 with C-11 and C-12 support the above deduction. In addition, the planar structure of **12** was similar to the known compound stachyline E except for the presence of an additional oxygenated methyl [15]. The recorded optical rotation for **12** was ${\left[\alpha \right]}_{D}^{25}$−4 (*c* 0.1, CH_3_OH), which has the same angle as that of stachyline E, ${\left[\alpha \right]}_{D}^{23}$−6 (*c* 0.1, CH_3_OH), suggesting that the configuration of C-11 in **12** is the same as that of stachyline E. Consequently, the structure of **12** was determined and assigned stachyline J (**12**). Compounds **10**–**12** may be separation artifacts.

By comparing their physicochemical properties and spectroscopic data with the reported literature values, other known compounds were determined. Compounds present in SCSIO 41050 were cordyanhydride A methyl ester (**2**) [10], cordyanhydride A (**3**) [12], 7-epiaustdiol (**6**) [14], 8-*O*-methylepiaustdiol (**7**) [14], 4-hydroxy-3,6-dimethyl-2*H*-pyran-2-one (**8**) [16], and 2,4-dihydroxy-3,6-dimethylbenzoic acid (**9**) [17]. Compounds present in SCSIO 41411 were stachylines G (**13**) and F (**14**) [15], (*E*)-4-(4-hydroxy-3-methylbut-2-enyloxy)benzaldehyde (**15**) [18], stachyline E (**16**) [15], penialidins C (**17**) [19], dibutylphthalate (**18**) [20], brefeldin G (**19**) [21], 9,12-octadecadieonic acid (**20**) [22], ɑ-linolenic acid (**21**) [23], linoleic acid (**22**) [24], and glycerol monlinoleate (**23**) [25].

### 2.2. Bioactive Assay

All the isolated compounds (**1**–**23**) were evaluated for cytotoxicity against four cancer cell lines (PC-3, 22Rv1, A549, WPMY-1), antibacterial activities against five bacteria (*Erysipelothrix rhusiopathiae* WH13013, *Streptococcus suis* SC19, *Escherichia coli* ATCC 25922, *Pseudomonas aeruginosa* ATCC 27853, *Staphylococcus aureus* ATCC 25923), and antifungal activities against five strains (*Botrytis cinerea*, *Verticillium dahlia* kieb., *Fusarium graminearum* schw., *Fusarium oxysporum* f.sp. *niveum*, *Rhizoctonia solani*). As shown in Table 3, maleicanhydridane (**4**), with one acid anhydride moiety, showed moderate cytotoxicity against cell lines A549 and WPMY-1, with IC_50_ values of 15.5 and 22.9 μM, respectively, while other compounds, including the cordyanhydride derivatives (**1**–**3**) with two acid anhydride moieties, were inactive (IC_50_ > 50 μM). In the antibacterial assay, only **9**, **21**, and **22** showed weak antibacterial activity against *S. suis*, with minimal inhibitory concentration (MIC) values of 50, 100, and 100 μg/mL, respectively. It is worth noting that three cordyanhydride derivatives (**1**–**3**) showed obvious antifungal activities, especially against *F. graminearum*, *F. oxysporum*, and *R. solani*, with MIC values of 6.25–12.5 μg/mL.

The obtained compounds were screened at 20 µM for their inhibitory activities against LPS-induced NF-κB activation in RAW264.7 cells, and no compounds showed obvious activities. The enzyme inhibitory activity assay was also conducted for acetylcholine esterase (AChE) and phosphodiesterase 4 (PDE4). PDE4 was involved in the regulation of proinflammatory cytokines via the degradation of cyclic adenosine monophosphate [26]. As a result, compounds **1**, **2**, **4**, and **6** at 10 μM displayed weak or moderate inhibition against PDE4 with inhibitory ratios of 49.7%, 27.5%, 11.2% and 39.6%, respectively. Meanwhile, **5**, **10**, and **11** at 50 μM displayed weak inhibition against AChE with inhibitory ratios of 21.3%, 22.3%, and 19.9%, respectively. Although the activities were weak, this is the first report of the AChE inhibitory activities of austdiol polyketides and stachyline derivates.

**Figure 4:**
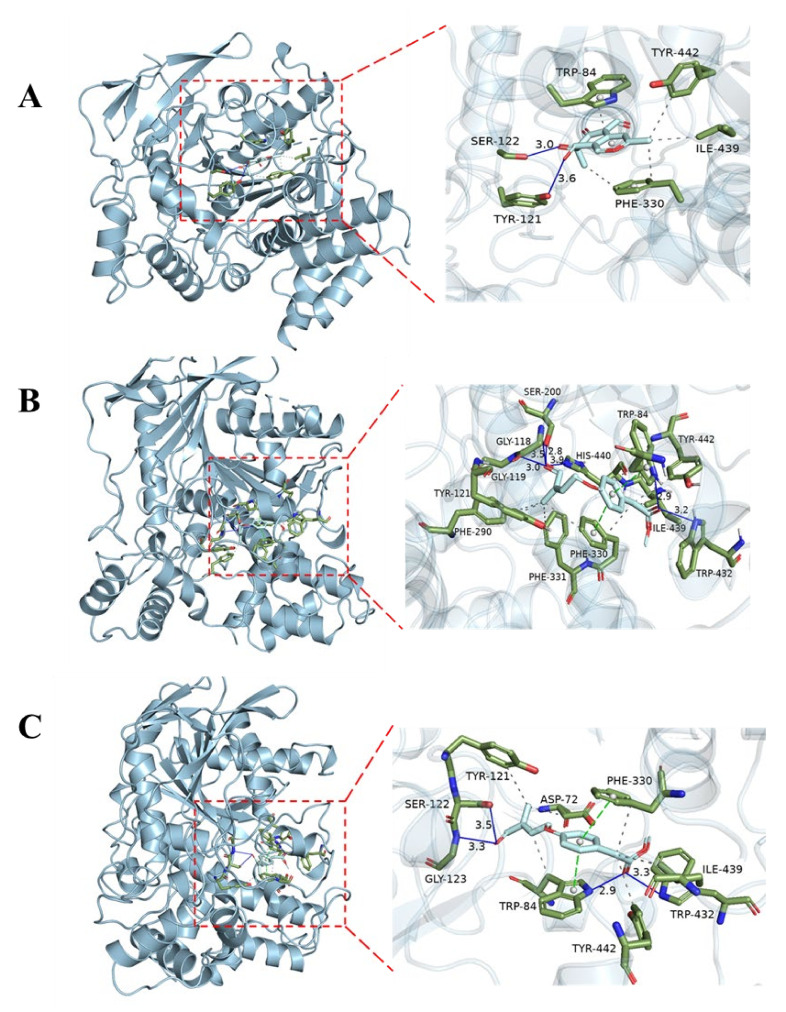
Molecular docking proposed binding interactions of compounds **5** (**A**), **10** (**B**), and **11** (**C**) with the active site residues of AChE (PDB ID: 1UT6). Blue solid line: hydrogen bond; black dotted line: hydrophobic interaction; green dotted line: π-π stacking interaction.

In order to further understand the interaction between the compounds and AChE protein, so as to improve the activity by structure optimization in the future, docking studies were carried out for **5**, **10,** and **11** in the active site of AChE (PDB: 1UT6) to gain insights into their molecular interactions. As a result, these ligands were favorably accommodated within the binding cleft with analogous anchoring conformations, exhibiting binding free energies (designated as S value) spanning from −8.7 to −8.4 kcal/mol. Compounds **5**, **10,** and **11** interacted with the AChE active site mainly through hydrogen bonds, π-π stacking contacts, and hydrophobic interactions (Figure 4). Compound **5** formed hydrogen bonds with amino acid residues TYR121 and SER122 within the target protein at distances of 3.6 Å and 3.0 Å, respectively. It also exhibited π-π stacking contacts with TRP84 and PHE330, as well as hydrophobic interactions with TRP84, PHE330, ILE439, and TYR442. Compound **10** established hydrogen bonds with TRP84, GLY118, GLY119, SER200, TRP432, and HIS440 at distances of 2.9 Å, 3.5Å, 3.0Å, 2.8Å, 3.2Å, and 3.9Å, respectively. It also showed π-π stacking contacts with TRP84 and PHE330, in addition to hydrophobic interactions with TRP84, TYR121, PHE290, PHE330, PHE331, ILE439, and TYR442. Compound **11** formed hydrogen bonds with TRP84, SER122, GLY123, and TRP43 at distances of 2.9 Å, 3.5Å, 3.3Å, and 3.3Å, respectively. It also exhibited π-π stacking contacts with TRP84 and PHE330, as well as hydrophobic interactions with ASP72, TRP84, TYR121, PHE330, ILE439, and TYR442. The binding of these compounds to the enzyme was stabilized through these interactions.

## 3. Materials and Methods

### 3.1. General Experimental Procedures

The UV spectrum was recorded on a Shimadzu UV-2600 PC spectrometer (Shimadzu, Beijing, China). The IR spectrum was obtained using an IR Affinity-1 spectrometer (Shimadzu). Optical rotations were determined with an Anton Paar MPC 500 polarimeter. HRESIMS spectra were recorded with a Bruker maXis Q-TOF mass spectrometer. The NMR spectra were recorded on a Bruker Avance-500 spectrometer (Bruker BioSpin International AG, Fällanden, Switzerland), and chemical shifts were recorded as *δ*-values. Semipreparative high-performance liquid chromatography (HPLC) was performed on the Hitachi Primaide with a DAD detector, using an ODS column (YMC-pack ODS-A, 10 × 250 mm, 5 μm). Thin-layer chromatography analysis (TLC) and column chromatography (CC) were carried out on plates precoated with silica gel GF254 (10–40 μm) and over silica gel (200–300 mesh) (Qingdao Marine Chemical Factory, Qingdao, China) and Sephadex LH-20 (Amersham Biosciences, Uppsala, Sweden), respectively. Spots were detected on TLC (Qingdao Marine Chemical Factory) under 254 nm UV light. All solvents employed were of analytical grade (Tianjin Fuyu Chemical and Industry Factory, Tianjin, China).

### 3.2. Fungal Material

The fungal strains SCSIO 41050 and SCSIO 41411 were isolated from a mangrove sediment sample, collected from Gaoqiao mangrove wetland (21.573°N, 109.767°E) in Zhanjiang, coastline of the northern part of Beibu Gulf, China. The strains were stored on MB agar (malt extract 15 g, sea salt 10 g, agar 16 g, H_2_O 1 L, pH 7.4–7.8) slants in liquefied petrolatum and deposited at Key Laboratory of Tropical Marine Bio-resources and Ecology, Chinese Academy of Sciences. The strains SCSIO 41050 and SCSIO 41411 were designated as *Talaromyces* sp. and *Penicillium* sp., due to their ITS sequences (GenBank accession No. OQ867300 and OQ052995) homology with those of *Talaromyces* sp. KT240143.1 and *Penicillium* sp. NR138263.1, respectively.

### 3.3. Fermentation and Extraction

The fungal strains were cultured in 200 mL seed medium (15 g malt extract, 10g sea salt, 1 L H_2_O) in 500 mL Erlenmeyer flasks at 28 °C for 3 days on a rotary shaker (180 rpm). Large-scale fermentations of SCSIO 41050 and SCSIO 41411 were incubated statically at 25 °C for 30 days using a rice medium (200 g rice, 2.5% sea salt, 230 mL H_2_O) in the 1 L flask (×60 and ×45, respectively). The fermented culture was extracted three times with EtOAc, yielding a reddish extract (130 g) and a brown extract (53.2 g), respectively.

### 3.4. Isolation and Purification

The SCSIO 41050 organic extract was subjected to silica gel CC using step gradient elution with petroleum ether/CH_2_Cl_2_ (0–100%, *v*/*v*) and CH_2_Cl_2_/CH_3_OH (0–100%, *v*/*v*) to obtain eight fractions (Frs. 1–8) based on TLC properties. Fraction 1 was subjected to semipreparative HPLC eluting with 88% CH_3_CN/H_2_O (0.4% TFA, 3 mL/min) to afford compound **4** (3.7 mg, *t*_R_ = 8.6 min). Fraction 2 was divided into four subfractions (Frs. 2-1–2-4) by semipreparative HPLC using step gradient elution with CH_3_CN/H_2_O (0.4% TFA, 65–85%, *v*/*v*, 0–30 min). Subfraction 2-1 was further purified by semipreparative HPLC (68% CH_3_CN/H_2_O (0.4% TFA), 2 mL/min) to afford **3** (18.5 mg, *t*_R_ = 18.2 min). Subfraction 2-3 was further purified by semipreparative HPLC (70% CH_3_CN/H_2_O (0.4% TFA), 2 mL/min) to afford **2** (40.5 mg, *t*_R_ = 20.5 min). **1** (11.6 mg, *t*_R_ = 26.2 min) was obtained from subfraction 2–4 by semipreparative HPLC, eluting with 70% CH_3_CN/H_2_O (0.4% TFA, 2 mL/min). Fraction 5 was divided into six subfractions (Frs. 5-1–5-4) by MPLC using step gradient elution with CH_3_OH/H_2_O (10–100%, *v*/*v*). Subfraction 5-1 was further purified by semipreparative HPLC (40% CH_3_OH/H_2_O (0.4% TFA), 2 mL/min) to afford **6** (92.6 mg, *t*_R_ = 9.7 min). Subfraction 5-3 was further divided by semipreparative HPLC (38% CH_3_OH/H_2_O (0.4% TFA), 2 mL/min) to afford **7** (3.4mg, *t*_R_ = 12.9 min), **5** (6.1 mg, *t*_R_ = 14.0 min), and **8** (40.8 mg, *t*_R_ = 15.7 min). **9** (26.6 mg, *t*_R_ = 17.5 min) was obtained from subfraction 5-4 by semipreparative HPLC, eluting with 53% CH_3_OH/H_2_O (0.4% TFA, 2 mL/min).

The SCSIO 41411 crude extract was chromatographed over an ODS RP-18 CC eluted with CH_3_OH/H_2_O (10–100%, *v*/*v*) to obtain fifteen fractions (Frs. 1–15). Fraction 2 was chromatographed over an ODS RP-18 CC eluted with CH_3_OH/H_2_O (10–100%, *v*/*v*) to obtain nine fractions (Fr. 2-1–Fr. 2-9). Fraction 2-8 was subjected to semipreparative HPLC, eluting with 62% CH_3_OH/H_2_O (2.5 mL/min) to afford **10** (4.0 mg, *t*_R_ = 15.6 min), **11** (5.5 mg, *t*_R_ = 13.8 min), and **17** (4.2 mg, *t*_R_ = 21.0 min). Fraction 2-5 was subjected to semipreparative HPLC, eluting with 31% CH_3_CN/H_2_O (3 mL/min) to afford **14** (24.0 mg, *t*_R_ = 9.5 min), **15** (1.3 mg, *t*_R_ = 14.0 min), and Fraction 2-5-2. Fraction 2-5-2 was further purified by semipreparative HPLC (55% CH_3_OH/H_2_O, 2 mL/min) to afford **12** (3.7 mg, *t*_R_ = 12.0 min) and **13** (1.6 mg, *t*_R_ = 9.7 min). Fraction 2-3 was subjected to semipreparative HPLC, eluting with 20% CH_3_CN/H_2_O (3 mL/min) to provide **16** (8.9 mg, *t*_R_ = 13.0 min) and **19** (11.4 mg, *t*_R_ = 16.5 min). Fraction 10 was subjected to semipreparative HPLC, eluting with 75% CH_3_CN/H_2_O (3 mL/min) to afford **18** (2.9 mg, *t*_R_ = 15.8 min). Fraction 11 was subjected to semipreparative HPLC, eluting with 90% CH_3_OH/H_2_O (3 mL/min) to give **20** (4.0 mg, *t*_R_ = 18.1 min) and Fraction 11-2. Further purification of Fraction 11-2 by HPLC (85% CH3CN/H_2_O, 3 mL/min) yielded **21** (23.6 mg, *t*_R_ = 14.1 min), **22** (106.4 mg, *t*_R_ = 20.0 min), and **23** (85.6 mg, *t*_R_ = 15.4 min).

### 3.5. Spectroscopic Data of New Compounds

Cordyanhydride A ethyl ester (**1**): yellow oil; **${\left[\alpha \right]}_{D}^{25}$** +2 (*c* 0.05, CH_3_OH); UV (CH_3_OH) *λ*_max_ (log *ε*) 205 (4.24), 250 (3.84), 320 (3.45) nm; IR (film) *ν*_max_ 2963, 2928, 2361, 1764, 1732, 1271, 1184, 1024, 974, 920 cm^−1^; ^1^H and ^13^C NMR data, Table 1; HRESIMS *m*/*z* 419.1701 [M + H]^+^ (calculated for C_22_H_27_O_8_^+^, 419.1700), 441.1521 [M + Na]^+^ (calculated for C_22_H_26_NaO_8_^+^, 441.1521).

Maleicanhydridane (**4**): yellow oil; ^1^H and ^13^C NMR data as shown in Table 1; HRESIMS *m*/*z* 253.1073 [M + H]^+^ (calculated for C_13_H_17_O_5_^+^, 253.1071).

Stachyline H (**10**): brown oil; UV (CH_3_OH) *λ*_max_ (log *ε*) 202 (3.81), 226 (3.51) nm; IR (film) *ν*_max_ 3446, 2949, 2879, 1732, 1611, 1510, 1435, 1223, 1159, 1003, 818 cm^−1^; ^1^H and ^13^C NMR data as shown in Table 2; HRESIMS *m*/*z* 273.1097 [M + Na]^+^ (calculated for C_14_H_18_NaO_4_^+^, 273.1097).

Stachyline I (**11**): brown oil; UV (CH_3_OH) *λ*_max_ (log *ε*) 202 (3.88), 226 (3.61) nm; IR (film) *ν*_max_ 3443, 2951, 2918, 1732, 1510, 1225, 1157, 1001, 820, cm^−1^; ^1^H and ^13^C NMR data as shown in Table 2; HRESIMS *m*/*z* 268.1540 [M + NH_4_]^+^ (calculated for C_14_H_22_NO_4_**^+^**, 268.1543) and 273.1091 [M + Na]^+^ (calculated for C_14_H_18_NaO_4_^+^, 273.1097).

Stachyline J (**12**): colorless oil; ${\left[\alpha \right]}_{D}^{25}$ −4 (*c* 0.1, CH_3_OH); UV (CH_3_OH) *λ*_max_ (log *ε*) 200 (4.36), 226 (3.90) nm; IR (film) *ν*_max_ 3446, 2976, 1734, 1514, 1246, 1163, 1032, 831, 806, 536 cm^−1^; ^1^H and ^13^C NMR data as shown in Table 2; HRESIMS *m*/*z* 286.1651 [M + NH_4_]^+^ (calculated for C_14_H_24_NO_5_^+^, 286.1649) and 291.1202 [M + Na]^+^ (calculated for C_14_H_20_NaO_5_^+^, 291.1203).

### 3.6. ECD Calculation of ***5***

Conformational analyses were carried out via Monte Carlo searching by means of Spartan’14 software (v1.1.4, Wavefunction, Irvine, CA, USA) using a Molecular Merck force field. The results showed ten lowest energy conformers within an energy window of 14 Kcal/mol. Then, these conformers were further re-optimized by the TD-DFT method at the B3LYP/6-31G(d) level in methanol using the Gaussian 16 program (A.03, Guassian, Pittsburgh, PA, USA) [27]. ECD calculations were further carried out at the B3LYP/6-311+G (d, p) level in methanol by adopting 50 excited states. The ECD spectra were generated based on Boltzmann distribution theory by SpecDis (1.70.1, SpecDis, Berlin, Germany) under a half band width of 0.3 eV and shifted by −25 nm to facilitate comparison to the experimental data.

### 3.7. Antibacterial and Antifungal Activity Assay

The antimicrobial activities against five bacteria (*Erysipelothrix rhusiopathiae* WH13013, *Streptococcus suis* SC19, *Escherichia coli* ATCC 25922, *Pseudomonas aeruginosa* ATCC 27853, *Staphylococcus aureus* ATCC 25923) and five fungi (*Botrytis cinerea*, *Verticillium dahlia* kieb., *Fusarium graminearum* schw., *Fusarium oxysporum* f.sp. *niveum*, *Rhizoctonia solani*) were evaluated using the methods described previously [9,28]. Streptomycin and penicillin were used as positive controls against bacteria, and cycloheximide was used against fungi.

### 3.8. Cytotoxicity Bioassay

Cytotoxicities against PC-3 (human prostate cancer cell line), 22Rv1 (human prostate cancer cell line), WPMY-1 (human prostatic stromal myofibroblast cell line), and A549 (human lung cancer cell), purchased from Shanghai Cell Bank, Chinese Academy of Sciences, were evaluated. Cell viability was analyzed by 3-(4,5)-dimethylthiahiazo (-z-yl)-3,5-di-phenytetrazoliumromide (MTT) assay as previously described [29]. In brief, cells were seeded in a 96-well plate at a density of 5 × 10^3^ per well overnight and treated with compounds for the required time. OD_570_ values were detected using a Hybrid Multi-Mode Reader (Synergy H1, BioTek, Santa Clara, CA, USA). The experiment was independently repeated three times.

### 3.9. NF-κB Bioassay

The suppression of LPS-induced NF-*κ*B activation in RAW264.7 cells was assessed using a luciferase reporter gene assay as detailed previously [30].

### 3.10. Enzyme Inhibitory Activities Assay

The protocols for expression, purification, and enzymatic assays of PDE4D2 were similar to those we described previously [26]. The inhibitory activity of AChE was assessed in vitro following a modified Ellman method [31].

### 3.11. Molecular Docking

The molecular docking simulation was implemented by utilizing the software AutoDock Tools (ADT 1.5.6) [32]. The crystal structure of AChE from *Tetronarce californica* (PDB ID: 1UT6) [33] was acquired from the Protein Data Bank (http://www.rcsb.org, accessed on 21 April 2005). The structures of ligands were generated in ChemBioOffice 18.0 (PerkinElmer Informatics, Waltham, MA, USA), followed by an MM2 calculation to minimize the conformation energy. The size of the grid box was 2.3 × 62.7 × 55.6, centered at *x*: 31.1, *y*: 27.7, *z*: 50.7. The other docking parameters, settings, and calculations were default, and the docking results were analyzed using the software PyMOL 2.4.0 (Schrödinger, New York, NY, USA).

## 4. Conclusions

In conclusion, the chemical investigation of the two mangrove-sediment fungal stains *Talaromyces* sp. SCSIO 41050 and *Penicillium* sp. SCSIO 41411 afforded 23 different compounds. Among these, cordyanhydride A ethyl ester (**1**) and stachylines H–J (**10**–**12**) were identified as new compounds, and maleicanhydridane (**4**) as a new natural product. Although some natural maleic anhydrides have been reported from *Talaromyces* species [34,35], the discovery of three cordyanhydride derivatives (**1**–**3**) with obvious antifungal activities was impressive. Other active natural products were also revealed, such as **4** with moderate cytotoxicity against cell lines A549 and WPMY-1, **1** and **6** with PDE4 inhibitory activities, and **5**, **10**, and **11** with potential for inhibiting AChE. The obtained results highlight the immense potential of the mangrove wetland ecosystem to yield novel natural products as well as bioactive compounds.

## Figures and Tables

**Table 1:** The NMR data of **1** and **4** (500 and 125 MHz, *δ* in ppm, DMSO-*d*_6_).

Pos.	1		4	
	*δ*_C_ Type	*δ*_H_ (*J* in Hz)	*δ*_C_ Type	*δ*_H_ (*J* in Hz)
1	171.5, C		171.4, C	
2	30.8, CH_2_	2.58 (dd, 9.5, 7.0)	31.0, CH_2_	2.59 (t, 7.5)
3	19.4, CH_2_	2.68 (t, 8.0)	18.9, CH_2_	2.75 (t, 7.5)
4	144.1, C		137.9, C	
5	143.0, C		136.8, C	
6	27.4, CH_2_	2.40 (m) 2.47 (overlapped)	117.0, CH	6.49 (dt, 16.0, 1.5)
7	37.6, CH	2.05 (m)	147.5, CH	7.05 (dt, 16.0, 6.5)
8	27.9, CH_2_	2.47 (overlapped)	26.6, CH_2_	2.29 (td, 7.1, 1.6)
9	138.1, C		12.5, CH_3_	1.05 (t, 7.5)
10	137.7, C		165.8, C	
11	117.0, CH	6.43 (dt, 15.5, 1.5)	164.6, C	
12	147.7, CH	7.08 (dt, 15.5, 6.5)	60.2, CH_2_	4.05 (q, 7.0)
13	26.6, CH_2_	2.28 (m)	14.0, CH_3_	1.17 (t, 7.0)
14	12.5, CH_3_	1.05 (t, 7.5)		
15	165.5, C			
16	166.0, C			
17	166.2, C			
18	164.5, C			
19	25.7, CH_2_	1.30 (m)		
20	10.7, CH_3_	0.88 (t, 7.5)		
21	60.3, CH_2_	4.05 (q, 7.0)		
22	14.0, CH_3_	1.17 (t, 7.0)		
7-OH				
8-OH				

**Table 2:** The NMR data of **10**–**12** (500 and 125 MHz, *δ* in ppm, DMSO-*d*_6_).

Pos.	10		11		12	
	*δ*_C_ Type	*δ*_H_ (*J* in Hz)	*δ*_C_ Type	*δ*_H_ (*J* in Hz)	*δ*_C_ Type	*δ*_H_ (*J* in Hz)
1	126.2, C		126.2, C		126.1, C	
2	130.3, CH	7.15 (d, 8.5)	130.3, CH	7.16 (d, 8.6)	130.4, CH	7.15 (d, 8.5)
3	114.5, CH	6.86 (d, 8.6)	114.5, CH	6.87 (d, 8.6)	114.4, CH	6.88 (d, 8.6)
4	157.3, C		157.3, C		157.9, C	
5	114.5, CH	6.86 (d, 8.6)	114.5, CH	6.87 (d, 8.6)	114.4, CH	6.88 (d, 8.6)
6	130.3, CH	7.15 (d, 8.5)	130.3, CH	7.16 (d, 8.6)	130.4, CH	7.15 (d, 8.5)
7	51.6, CH_2_	3.58 (s)	51.6, CH_2_	3.59 (overlapped)	39.3, CH_2_	3.58 (s)
8	171.9, C		171.9, C		172.0, C	
9	39.8, CH_3_	3.59 (s)	39.2, CH_3_	3.59 (overlapped)	51.6, CH_3_	3.59 (s)
10	63.6, CH_2_	4.57 (d, 6.6)	64.0, CH_2_	4.57 (d, 6.5)	69.8, CH_2_	4.19 (dd, 10.1, 2.3) 3.76 (dd, 10.0, 8.0)
11	121.1, CH	5.43 (t, 6.3)	118.1, CH	5.63 (t, 6.3)	75.8, CH	3.52 (d, 7.9)
12	140.4, C		140.5, C		70.9, C	
13	59.8, CH_2_	4.00 (s)	65.4, CH_2_	3.84 (s)	27.4, CH_3_	1.13 (s)
14	21.0, CH_3_	1.75 (s)	13.8, CH_3_	1.65 (s)	24.3, CH_3_	1.07 (s)
13-OH				4.87 (s)		
12-OH						4.40 (s)
11-OH						4.98 (s)

**Table 3:** Cytotoxic, antibacterial and antifungal activities of isolated compounds.

	Tested Compounds					
Cells (IC_50_, μM)	4	Others				Positive
PC-3	>50	>50				0.12 ^a^
22Rv1	>50	>50				0.03 ^a^
A549	15.5	>50				29.95 ^a^
WPMY-1	22.9	>50				0.51 ^a^
**Bacteria (MIC, μg/mL)**	**9**	**21**	**22**	**Others**		**Positive**
*E* *. rhusiopathiae*	>100	>100	100	>100		12.5 ^b^
*S. suis*	50	100	100	>100		12.5 ^b^
*E.* *coli*	>100	>100	>100	>100		50 ^c^
*P. aeruginosa*	>100	>100	>100	>100		12.5 ^b^
*S. aureus*	100	>100	>100	>100		12.5 ^b^
**Fungi (MIC, μg/mL)**	**1**	**2**	**3**	**9**	**Others**	
*B. cinerea*	12.5	25	>100	>100	>100	12.5 ^d^
*V. dahlia*	100	>100	>100	50	>100	12.5 ^d^
*F. graminearum*	6.25	12.5	12.5	50	>100	12.5 ^d^
*F. oxysporum*	6.25	6.25	12.5	100	>100	25 ^d^
*R. solani*	6.25	6.25	12.5	>100	>100	25 ^d^

^a^ docetaxel; ^b^ penicillin; ^c^ streptomycin; ^d^ cycloheximide.

## Data Availability

The data presented in this study are available on request from the corresponding author.

## Supplementary Materials

The following supporting information can be downloaded at: https://www.mdpi.com/article/10.3390/md21060327/s1, Figures S1–S39: NMR, HRESIMS, UV, IR and CD spectra of compounds **1**, **4**, and **10**–**12**; Tables S1 and S2: ECD calculation details for **5**; The spectroscopic data of **2**–**3**, **5**–**9**, and **13**–**23**; ITS sequence data of the strains.
