# Supplementary material for: Bioactive Polyketides and Benzene Derivatives from Two Mangrove Sediment-Derived Fungi in the Beibu Gulf
Source: Mar Drugs. 2023 May 26;21(6):327. doi: 10.3390/md21060327 (PMC10304204; doi:10.3390/md21060327)
Supplement: Supplementary file 1 [file marinedrugs-21-00327-s001.zip › marinedrugs-2416154-supplementary.pdf]

Supporting Information

Bioactive Polyketides and Benzene Derivatives from Two Mangrove Sediment-Derived Fungi in the Beibu Gulf

Bo Peng [1,†], Jian Cai [2,†], Zimin Xiao [3], Manli Liu [4], Xinlong Li [2], Bin Yang [2], Wei Fang [4], Yi-You Huang [5], Chunmei Chen [2,*], Xuefeng Zhou [2,*], Huaming Tao [3,*]

[1] Institute for Environmental and Climate Research, Jinan University, Guangzhou, 511443, China; pengbo@jnu.edu.cn

[2] CAS Key Laboratory of Tropical Marine Bio-resources and Ecology/ Guangdong Key Laboratory of Marine Materia Medica, South China Sea Institute of Oceanology, Chinese Academy of Sciences, Guangzhou 510301, China; caijian19@mails.ucas.ac.cn (J.C.); 13409379936@163.com (X.L.); yangbin@scsio.ac.cn (B.Y.)

[3] Guangdong Provincial Key Laboratory of Chinese Medicine Pharmaceutics, School of Traditional Chinese Medicine, Southern Medical University, Guangzhou 510515, China; 15917491112@163.com (Z.X.)

[4] Hubei Biopesticide Engineering Research Center, Hubei Academy of Agricultural Science, Wuhan 430064, China; manli.liu@nberc.com (M.L.); wei.fang@nberc.com (W.F.)

[5] Key Laboratory of Tropical Biological Resources of Ministry of Education, School of Pharmaceutical Sciences, Hainan University, Haikou, 570228, China. hyyou@hainanu.edu.cn (Y.H.)

* Correspondence: chenchunmei18@mails.ucas.ac.cn (C.C.); xfzhou@scsio.ac.cn (X.Z.); taohm@smu.edu.cn (H.T.)

† These authors contributed equally to this work.

Table of Contents

The physicochemical data of the known compounds 2, 3, 6–9, and 13–23. 4

Figure S1. ^{1}H NMR spectrum of cordyanhydride A ethyl ester (**1**) in DMSO-d_6. 9

Figure S2. ^{13}C NMR spectrum of cordyanhydride A ethyl ester (**1**) in DMSO-d_6. 9

Figure S3. HSQC spectrum of cordyanhydride A ethyl ester (**1**) in DMSO-d_6. 10

Figure S4. HMBC spectrum of cordyanhydride A ethyl ester (**1**) in DMSO-d_6. 10

Figure S5. ^{1}H–^{1}H COSY spectrum of cordyanhydride A ethyl ester (**1**) in DMSO-d_6. 11

Figure S6. HRESIMS spectrum of cordyanhydride A ethyl ester (**1**). 12

Figure S7. IR spectrum of cordyanhydride A ethyl ester (**1**). 12

Figure S8. UV spectrum of cordyanhydride A ethyl ester (**1**) in MeOH. 13

Figure S9. ^{1}H NMR spectrum of stachyline H (**10**) in DMSO-d_6. 13

Figure S10. ^{13}C NMR spectrum of stachyline H (**10**) in DMSO-d_6. 14

Figure S11. HSQC spectrum of stachyline H (**10**) in DMSO-d_6 14

Figure S12. HMBC spectrum of stachyline H (**10**) in DMSO-d_6. 15

Figure S13. ^{1}H–^{1}H COSY spectrum of stachyline H (**10**) in DMSO-d_6. 15

Figure S14. NOESY spectrum of stachyline H (**10**) in DMSO-d_6. 16

Figure S15. HRESIMS spectrum of stachyline H (**10**). 17

Figure S16. IR spectrum of stachyline H (**10**). 17

Figure S17. UV spectrum of stachyline H (**10**) in MeOH. 18

Figure S18. ^{1}H NMR spectrum of stachyline I (**11**) in DMSO-d_6. 18

Figure S19. ^{13}C NMR spectrum of stachyline I (**11**) in DMSO-d_6. 19

Figure S20. HSQC spectrum of stachyline I (**11**) in DMSO-d_6. 19

Figure S21. HMBC spectrum of stachyline I (**11**) in DMSO-d_6. 20

Figure S22. ^{1}H–^{1}H COSY spectrum of stachyline I (**11**) in DMSO-d_6. 20

Figure S23. NOESY spectrum of stachyline I (**11**) in DMSO-d_6. 21

Figure S24. HRESIMS spectrum of stachyline I (**11**). 22

Figure S25. IR spectrum of stachyline I (**11**). 22

Figure S26. UV spectrum of stachyline I (**11**) in MeOH. 23

Figure S27. ^{1}H NMR spectrum of stachyline J (**12**) in DMSO-d_6. 23

Figure S28. ^{13}C NMR spectrum of stachyline J (**12**) in DMSO-d_6. 24

Figure S29. HSQC spectrum of stachyline J (**12**) in DMSO-d_6. 24

Figure S30. HMBC spectrum of stachyline J (**12**) in DMSO-d_6. 25

Figure S31. ^{1}H–^{1}H COSY spectrum of stachyline J (**12**) in DMSO-d_6. 25

Figure S32. NOESY spectrum of stachyline J (**12**) in DMSO-d6. 26

Figure S33. HRESIMS spectrum of stachyline J (**12**). 27

Figure S34. IR spectrum of stachyline J (**12**). 27

Figure S35. UV spectrum of stachyline J (**12**) in MeOH. 28

Figure S36. ECD spectrum of stachyline J (**12**) in MeOH. 29

Figure S37. ^{1}H NMR spectrum of maleicanhydridane (**4**) in DMSO-d_6. 29

Figure S38. 13C NMR spectrum of maleicanhydridane (**4**) in DMSO-d6. 29

Figure S39. HRESIMS spectrum of maleicanhydridane (**4**). 30

ECD Calculation Details for 5. 30

Table S1. Energies of **5** at MMFF94 force field. 31

Table S2. Energies of **5** at B3LYP/6–31+g(d) level in methanol. 31

The strain's (*Talaromyces* sp. SCSIO 41050) ITS sequence of the rDNA 32

The strain's (*Penicillium* sp. SCSIO 41411) ITS sequence of the rDNA 32

The physicochemical data of the known compounds 2, 3, 6–9, and 13–23.

Cordyanhydride A methyl ester (**2**): Yellow oil; ^{1}H NMR (500 MHz, DMSO-d_6) δ_H 7.30 (1H, dt, J = 16.0, 6.5 Hz, H-12), 6.65 (1H, dt, J = 16.0, 1.5 Hz, H-11), 3.81 (3H, s, OCH_3-1), 2.91 (2H, t, J = 7.0 Hz, H-3), 2.82 (2H, dd, J = 7.5, 6.5 Hz, H_2-2), 2.71 (2H, ddd, J = 12.5, 7.5, 2.5 Hz, H-6), 2.63 (1H, dt, J = 14.0, 7.0 Hz, a8-H), 2.50 (2H, pd, J = 7.5, 1.5 Hz, H-13), 2.28 (1H, hept, J = 6.5 Hz, βH-8), 1.52 (2H, p, J = 7.0 Hz, H-19), 1.27 (3H, t, J = 7.5 Hz, H-14), 1.10 (3H, t, J = 7.5 Hz, H-20); ^{13}C NMR (125 MHz, DMSO-d_6) δ_C 171.9 (C-1), 166.1 (C-17), 166.0 (C-16), 165.5 (C-15), 164.5 (C-18), 147.7 (C-12), 144.0 (C-5), 143.1 (C-4), 138.1 (C-10), 137.6 (C-9), 116.9 (C-11), 51.6 (-OCH_3), 37.6 (C-7), 30.6 (C-2), 27.9 (C-6), 27.3 (C-8), 26.6 (C-13), 25.7 (C-19), 19.4 (C-3), 12.4 (C-14), 10.5 (C-20).

Cordyanhydride A (**3**): Yellow oil; ^{1}H NMR (500 MHz, DMSO-d_6) δ_H 7.08 (1H, dt, J = 15.4, 6.3 Hz, H-12), 6.43 (1H, dt, J = 15.4, 1.4 Hz, H-11), 2.65 (2H, t, J = 7.7 Hz, H_2-3), 2.52 (4H, m, H-2/αH-6/αH-8), 2.41 (1H, dt, J = 14.0, 6.3 Hz, βH-6), 2.28 (2H, pd, J = 7.7, 1.4 Hz, H-13), 2.06 (1H, p, J = 7.0 Hz, βH-8), 1.30 (2H, p, J = 7.0 Hz, H-19), 1.05 (3H, t, J = 7.7 Hz, H-14), 0.88 (3H, t, J = 7.7 Hz, H-20); ^{13}C NMR (125 MHz, DMSO-d_6) δ_C 173.0 (C-1), 166.2 (C-17), 166.1 (C-16), 165.6 (C-15), 164.5 (C-18), 147.8 (C-12), 144.4 (C-5), 143.0 (C-4), 138.1 (C-10), 137.7 (C-9), 117.0 (C-11), 37.7 (C-7), 31.0 (C-2), 28.0 (C-6), 27.5 (C-8), 26.7 (C-13), 25.8 (C-19), 19.6 (C-3), 12.5 (C-14), 10.6 (C-20).

Austdiol (**5**): Yellow powder, $[\alpha]_D^{25}$ +23 (c 0.05, CH_3OH); UV (CH_3OH) λ_{max} (log ε) 200 (3.61), 256 (3.71), 380 (3.93) nm; IR (film) ν_{max} 3392, 2920, 1681, 1471, 1199, 1138, 1045 cm^{-1}; ^{1}H NMR (500 MHz, DMSO-d_6) δ_H 10.00 (1H, s, H-12), 8.20 (1H, s, H-1), 8.19 (1H, s, H-4), 5.93 (1H, brs, 8-OH), 5.23 (1H, brs, 7-OH), 4.39 (1H, s, H-8), 2.42 (3H, s, H-11), 1.09 (3H, s, H-13); ^{13}C NMR (125 MHz, DMSO-d_6) δ_C 198.3 (C-6), 189.4 (CHO-12), 166.3 (C-3), 151.9 (C-1), 148.7 (C-10), 122.2 (C-9), 108.2 (C-5), 106.4 (C-4), 74.3 (C-7), 70.6 (C-8), 18.8 (C-13), 19.9 (C-11); HRESIMS m/z 237.0765 $[M + H]^+$ (calcd for $C_{12}H_{13}O_5^+$, 237.0757), 259.0581 $[M + Na]^+$ (calcd for $C_{12}H_{12}NaO_5^+$, 259.0577).

7-epiaustdiol (**6**): Yellow powder, $[\alpha]_D^{25}$ +172 (*c* 0.05, CH_3OH); ^1H NMR (700 MHz, DMSO-d_6) δ_H 9.98 (1H, s, H-12), 8.29 (1H, s, H-1), 8.20 (1H, s, H-4), 4.31 (1H, s, H-8), 2.42 (3H, s, H-11), 1.14 (3H, s, H-13); ^{13}C NMR (175 MHz, DMSO-d_6) δ_C 198.3 (C-6), 189.2 (CHO-12), 166.0 (C-3), 152.7 (C-1), 148.0 (C-10), 121.5 (C-9), 108.2 (C-5), 106.7 (C-4), 74.5 (C-7), 71.5 (C-8), 21.9 (C-13), 20.0 (C-11).

8-*O*-methylepiaustdiol (**7**): Yellow powder, $[\alpha]_D^{25}$ +160 (*c* 0.05, CH_3OH); ^1H NMR (500 MHz, DMSO-d_6) δ_H 9.96 (1H, s, H-12), 8.44 (1H, s, H-4), 8.18 (1H, s, H-1), 3.96 (1H, s, H-1), 3.18 (3H, s, H-14), 2.41 (3H, s, H-11), 1.11 (3H, s, H-13); ^{13}C NMR (125 MHz, DMSO-d_6) δ_C 198.5 (C-6), 189.1 (CHO-12), 166.2 (C-3), 152.7 (C-1), 147.5 (C-10), 117.2 (C-9), 108.4 (C-5), 106.8 (C-4), 81.1 (C-8), 74.2 (C-7), 56.8 (C-14), 23.2 (C-13), 20.0 (C-11).

4-Hydroxy-3,6-dimethyl-2*H*-pyran-2-one (**8**): White powder, ^1H NMR (500 MHz, DMSO-d_6) δ_H 11.08 (1H, s, 3-OH), 5.98 (1H, s, H-4), 2.13 (3H, s, H-6), 1.73 (3H, s, H-7); ^{13}C NMR (125 MHz, DMSO-d_6) δ_C 165.2 (C-1), 165.0 (C-3), 159.4 (C-5), 99.8 (C-4), 96.4 (C-2), 19.3 (C-6), 8.4 (C-7).

2,4-Dihydroxy-3,6-dimethylbenzoic acid (**9**)： Red powder, ^1H NMR (700 MHz, DMSO-d_6) δ_H 13.44 (1H, s, 1-COOH), 12.62 (1H, s, 2-OH), 10.04 (1H, s, 4-OH), 6.25 (1H, s, H-5), 2.39 (3H, s, H-9), 1.93 (3H, s, H-8); ^{13}C NMR (175 MHz, DMSO-d_6) δ_C 174.2 (C-7), 163.1 (C-2), 160.0 (C-4), 139.6 (C-6), 110.3 (C-5), 107.9 (C-1), 103.5 (C-3), 23.8 (C-9), 8.0 (C-8).

Stachylines G (**13**): Colorless oil; ^1H NMR (700 MHz, DMSO-d_6) δ_H 7.12 (2H, d, J = 8.0 Hz, H-2, 6), 6.80 (2H, d, J = 8.1 Hz, H-3, 5), 5.42 (1H, m, H-2'), 4.55 (2H, d, J = 6.4 Hz, H-1), 3.99 (2H, s, H-4'), 3.31 (2H, s, H-7), 1.75 (3H, s, H-5'); ^{13}C NMR (175 MHz, DMSO-d_6) δ_C 174.2 (C-8), 156.58 (C-4), 140.24 (C-3'), 130.21 (C-2), 130.21 (C-6), 129.27 (C-1), 121.31 (C-2'), 114.05 (C-3), 114.05 (C-5), 63.57 (C-1'), 59.83 (C-4'), 42.24 (C-7), 20.99 (C-5).

Stachylines F (**14**): White powder; ^1H NMR (500 MHz, DMSO-d_6) δ_H 7.15 (2H, d, J = 8.5 Hz, H-2, 6), 6.87 (2H, d, J = 8.5 Hz, H-3, 5), 5.64 (1H, q, J = 5.4 Hz, H-2'), 4.57

(2H, d, J = 6.5 Hz, H-1'), 3.85 (2H, s, H-4'), 3.47 (2H, s, H-7), 1.65 (3H, s, H-5'); ^{13}C NMR (125 MHz, DMSO-d_6) δ_C 173.1 (C-8), 157.2 (C-4), 140.5 (C-3'), 130.4 (C-2), 130.4 (C-6), 127.0 (C-1), 118.2 (C-2'), 114.4 (C-3), 114.4 (C-5), 65.5 (C-1'), 64.0 (C-4'), 40.0 (C-7), 13.8 (C-5').

(*E*)-4-(4-Hydroxy-3-methylbut-2-enyloxy)benzaldehyde (**15**): Colorless oil; ^{1}H NMR (700 MHz, DMSO-d_6) δ_H 9.86 (1H, s, 1-CHO), 7.86 (2H, d, J = 8.7 Hz, H-2, 6), 7.13 (2H, d, J = 8.7 Hz, H-3, 5), 5.66 (1H, td, J=6.6, 1.4 Hz, H-2'), 4.72 (2H, d, J = 6.7 Hz, H-1'), 3.85 (2H, s, H-4'), 1.67 (3H, s, H-5'); ^{13}C NMR (175 MHz, DMSO-d_6) δ_C 191.3 (1-CHO), 163.5 (C-4), 141.5 (C-3'), 131.8 (C-2), 131.8 (C-6), 129.6 (C-1), 117.1 (C-2'), 115.1 (C-3), 115.1 (C-5), 65.3 (C-4'), 64.7 (C-1'), 13.8 (C-5').

Stachyline E (**16**): Colorless oil; ^{1}H NMR (500 MHz, DMSO-d_6) δ_H 7.14 (2H, d, J = 8.3 Hz, H-2, 6), 6.86 (2H, d, J = 8.5 Hz, H-3, 5), 4.19 (1H, dd, J = 10.1, 2.2 Hz, H-1'a), 3.76 (1H, dd, J = 10.0, 8.1 Hz, H-1'b), 3.52 (1H, dd, J = 8.1, 2.2 Hz, H-2'), 3.45 (2H, s, H-7), 1.13 (3H, s, H-4'), 1.07 (3H, s, H-5'); ^{13}C NMR (125 MHz, DMSO-d_6) δ_C 173.2 (C-8), 157.6 (C-4), 130.3 (C-2, 6), 127.1 (C-1), 114.3 (C-3, 5), 75.8 (C-2'), 70.8 (C-3'), 69.8 (C-1'a), 40.1 (C-7), 27.4 (C-4'), 24.3 (C-5').

Penialidins C (**17**): Yellow powder; ^{1}H NMR (500 MHz, DMSO-d_6) δ_H 6.86 (1H, s, H-13), 5.69 (1H, s, H-3), 5.15 (2H, s, H-6), 2.00 (3H, s, H-1); ^{13}C NMR (125 MHz, DMSO-d_6) δ_C 171.0 (C-7), 169.0 (C-14), 167.3 (C-2), 158.3 (C-4), 150.0 (C-12), 144.9 (C-9), 144.1 (C-11), 117.3 (C-10), 112.2 (C-8), 103.1 (C-13), 101.1 (C-5), 94.4 (C-3), 64.1 (C-6), 19.9 (C-1).

Dibutylphthalate (**18**): Colorless oil; ^{1}H NMR (500 MHz, DMSO-d_6) δ_H 7.73 (2H, dt, J=7.3, 3.7 Hz, H-2, 5), 7.68 (2H, dd, J=5.7, 3.3 Hz, H-3, 4); dibutyl protons: 4.24 (4H, t, J=6.5 Hz), 1.65 (4H, m), 1.39 (4H, m), 0.93 (6H, t, J=7.4 Hz, 2CH_3); ^{13}C NMR (125 MHz, DMSO) δ_C 167.5 (C-1), 132.2(C-2), 132.1 (C-3), 129.2 (C-4), 65.6 (C-5), 30.5 (C-6), 19.2 (C-7), 14.1 (C-8).

Brefeldin G (**19**): White powder; ^{1}H NMR (500 MHz, DMSO-*d*$_6$) δ_H 6.73 (1H, dd, *J*=15.5, 4.5 Hz, H-3), 5.84 (1H, d, *J*=15.4 Hz, H-2), 5.32 (2H, m, H-10, 11), 4.07 (1H, d, *J*=4.7 Hz, H-4), 4.01 (1H, dd, *J*=6.4, 4.4 Hz, H-7), 2.39 (2H, t, *J*=7.3 Hz, H-14), 2.33 (1H, d, *J*=8.4 Hz, H-9), 2.06 (3H, s, H-16), 1.99 (1H, m, H-5), 1.90 (2H, m, H-12), 1.84 (1H, m, H-6a), 1.64 (1H, m, H-13a), 1.50 (2H, m, H-6b, 13b), 1.27-1.35 (2H, m, H-8). ^{13}C NMR (125 MHz, DMSO-*d*$_6$)) δ_C 208.5 (C-15), 167.8 (C-1), 150.9 (C-3), 135.1 (C-11), 128.8 (C-10), 120.7 (C-2), 70.4 (C-7), 69.0 (C-4), 48.5 (C-5), 42.6 (C-9), 42.5 (C-8), 42.0 (C-14), 35.1 (C-6), 31.3 (C-12), 29.8 (C-16), 23.1 (C-13).

9,12-octadecadieonic acid (**20**): ^{1}H NMR (500 MHz, Chloroform-*d*) δ_H 5.36 (4H, m, H-9, 10, 12, 13), 2.77 (2H, t, *J* =6.6 Hz, H-11), 2.35 (2H, t, *J* =7.5 Hz, H-2), 2.05 (4H, q, *J* =6.9 Hz, H-8, 14), 1.63 (2H, m, H-3), 1.33 (14H, m, H-4, 5, 6, 7, 15, 16, 17), 0.89 (3H, t, *J*= 6.5 Hz, H-18); ^{13}C NMR (125 MHz, Chloroform-*d*) δ_C 178.7 (C-1), 130.4 (C-9), 130.2 (C-10), 128.2 (C-12), 128.1 (C-13), 33.9, 31.7, 29.7, 29.5, 29.3, 29.2, 29.2, 27.4, 27.3, 25.8, 24.8, 22.7, 14.2.

α-linolenic acid (**21**): ^{1}H NMR (500 MHz, Chloroform-*d*) δ_H 5.45 (6H, m, H-9, 10, 12, 13, 15, 16), 2.81 (4H, t, *J* =6.0 Hz, H-11, 14), 2.34 (2H, t, *J* =7.5 Hz, H-2), 2.07 (4H, m, H-8, 17), 1.63 (2H, m, H-3), 1.33 (10H, d, *J* =9.4 Hz, H-4, 5, 6, 7), 0.97 (3H, t, *J* =7.5 Hz, H-18); ^{13}C NMR (125 MHz, Chloroform-*d*) δ_C 180.1 (C-1), 132.1 (C-9), 130.4 (C-10), 128.4 (C-12), 128.4 (C-13), 127.9 (C-15), 127.3 (C-16), 34.2 (C-2), 29.7 (C-4), 29.3 (C-5), 29.2 (C-6), 29.2 (C-7), 27.3 (C-3), 25.8 (C-8), 25.7 (C-11), 24.8 (C-14), 20.7 (C-17), 14.4 (C-18).

Linoleic acid (**22**); ^{1}H NMR (500 MHz, Chloroform-*d*) δ_H 11.30 (1H, s, -COOH), 5.35 (4H, ddq, *J* =14.0, 7.1, 4.3, 3.4 Hz, H-9, 10, 12, 13), 2.77 (2H, t, *J* =6.5 Hz, H-11), 2.34 (2H, t, *J* =7.5 Hz, H-2), 2.05 (4H, q, *J* =6.8 Hz, H-8, 14), 1.63 (2H, m, H-3), 1.32 (14H, m, H-4, 5, 6, 7, 15, 16, 17), 0.89 (3H, t, *J* =6.8 Hz, H-18); ^{13}C NMR (125 MHz, Chloroform-*d*) δ_C 180.7 (C-1), 130.3 (C-9), 130.1 (C-10), 128.2 (C-12), 128.0 (C-13), 34.3 (C-2), 31.7 (C-4), 29.7 (C-5), 29.5 (C-6), 29.3 (C-7), 29.2 (C-15), 29.2 (C-16), 27.3 (C-8), 27.3 (C-14), 25.8 (C-11), 24.8 (C-3), 22.7 (C-17), 14.2 (C-18).

Glycerol monlinoleate (**23**): ^{1}H NMR (500 MHz, Chloroform-*d*) δ_H 5.33 (4H, m, H-9', 10', 12', 13'), 4.12 (2H, m, H-1), 3.89 (1H, m, H-2), 3.66 (1H, dd, *J*=11.6, 3.7 Hz, H-3a), 3.56 (1H, dd, *J*=11.6, 6.0 Hz, H-3b), 2.75 (2H, t, *J*=6.7 Hz, H-11'), 2.32 (2H, t, *J*=7.6 Hz, H-2'), 2.03 (4H, q, *J*=7.0 Hz, H-8', 14'), 1.60 (2H, t, *J*=7.3 Hz, H-3'), 1.30 (14H, m, 4'~7', H-15'~17'), 0.87 (3H, t, *J*=6.8 Hz, H-18'); ^{13}C NMR (125 MHz, $CDCl_3$) δ_C 174.4 (C-1'), 130.3 (C-9'), 130.0 (C-13'), 128.1 (C-10'), 127.9 (C-12'), 70.3 (C-2), 65.1 (C-1), 63.4 (C-3), 34.2 (C-2'), 31.6 (C-14'), 29.6 (C-16'), 29.4 (C-7'), 29.2 (C-8'), 29.2 (C-2',15'), 27.2 (4', 5'), 25.7 (C-6'), 24.9 (C-8'), 22.6 (C-17'), 14.1 (C-18').

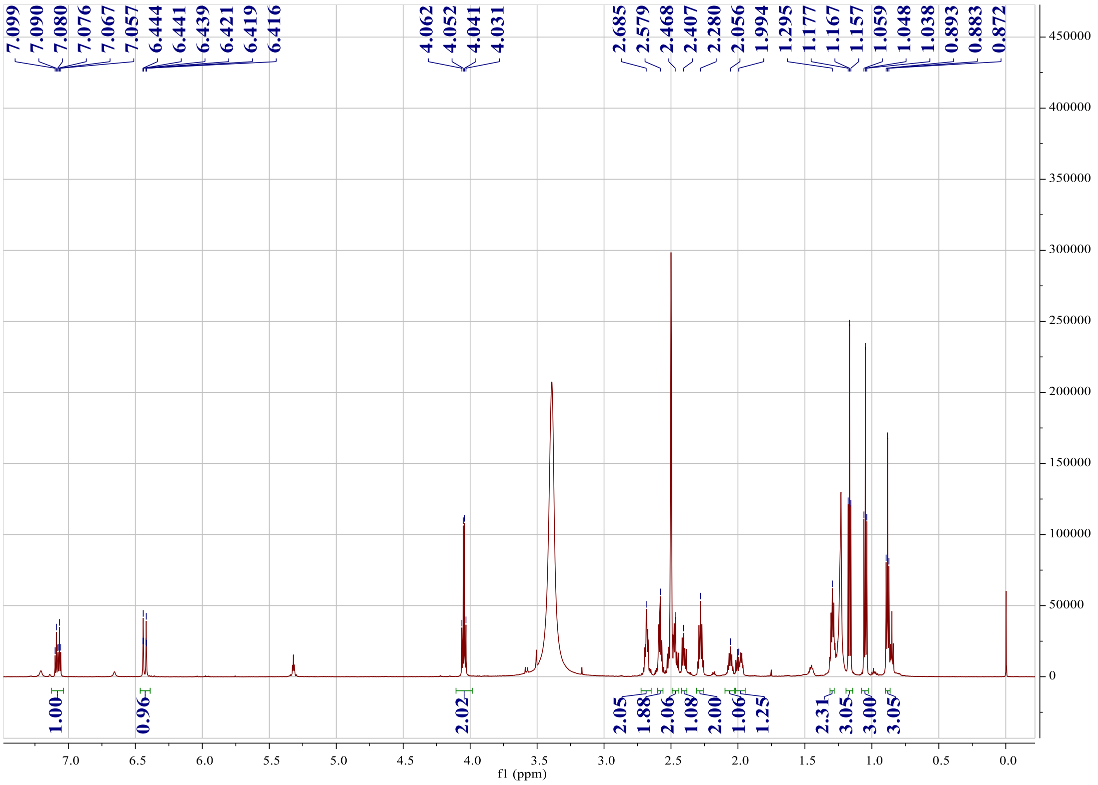

Figure S1. ^{1}H NMR spectrum of cordyanhydride A ethyl ester (**1**) in DMSO-d_6.

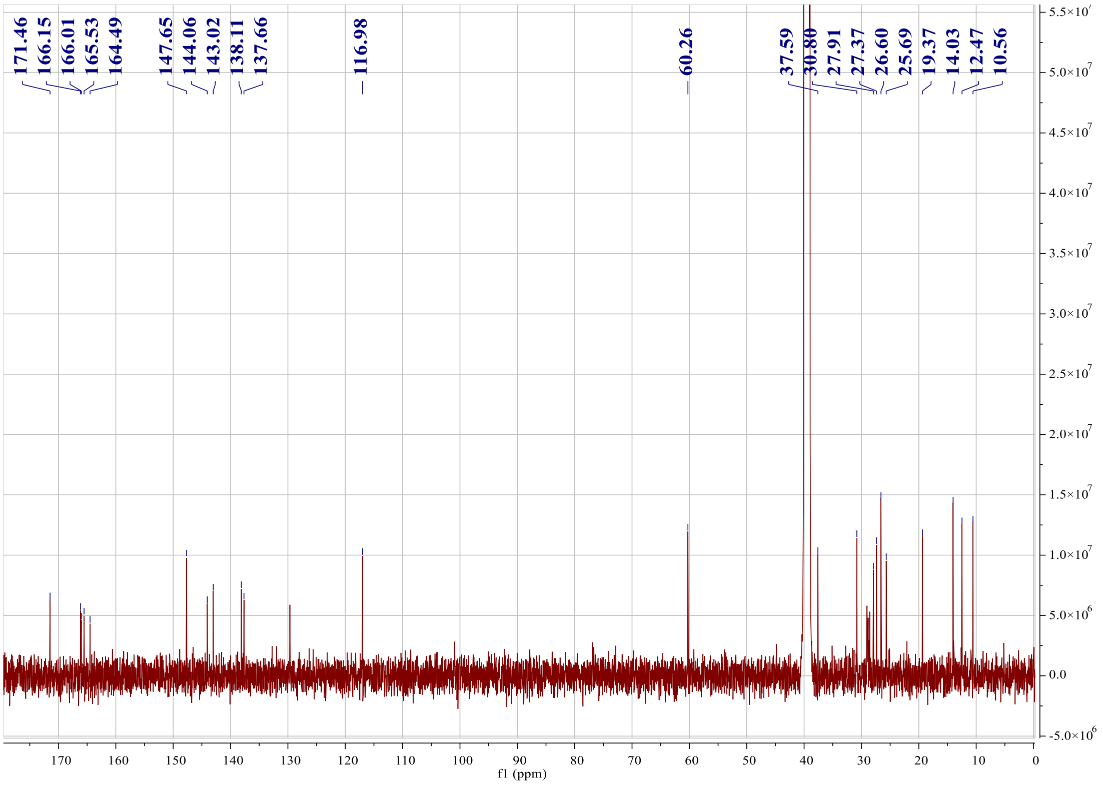

Figure S2. ^{13}C NMR spectrum of cordyanhydride A ethyl ester (**1**) in DMSO-d_6.

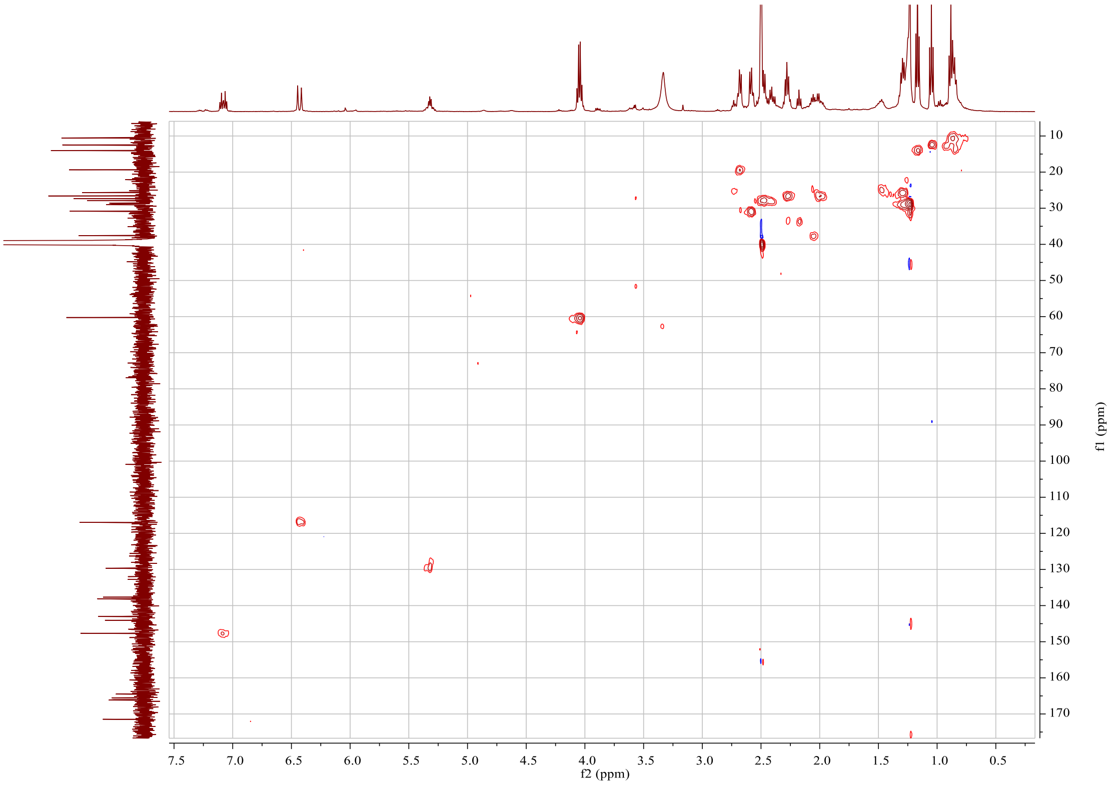

Figure S3. HSQC spectrum of cordyanhydride A ethyl ester (**1**) in DMSO-d_6.

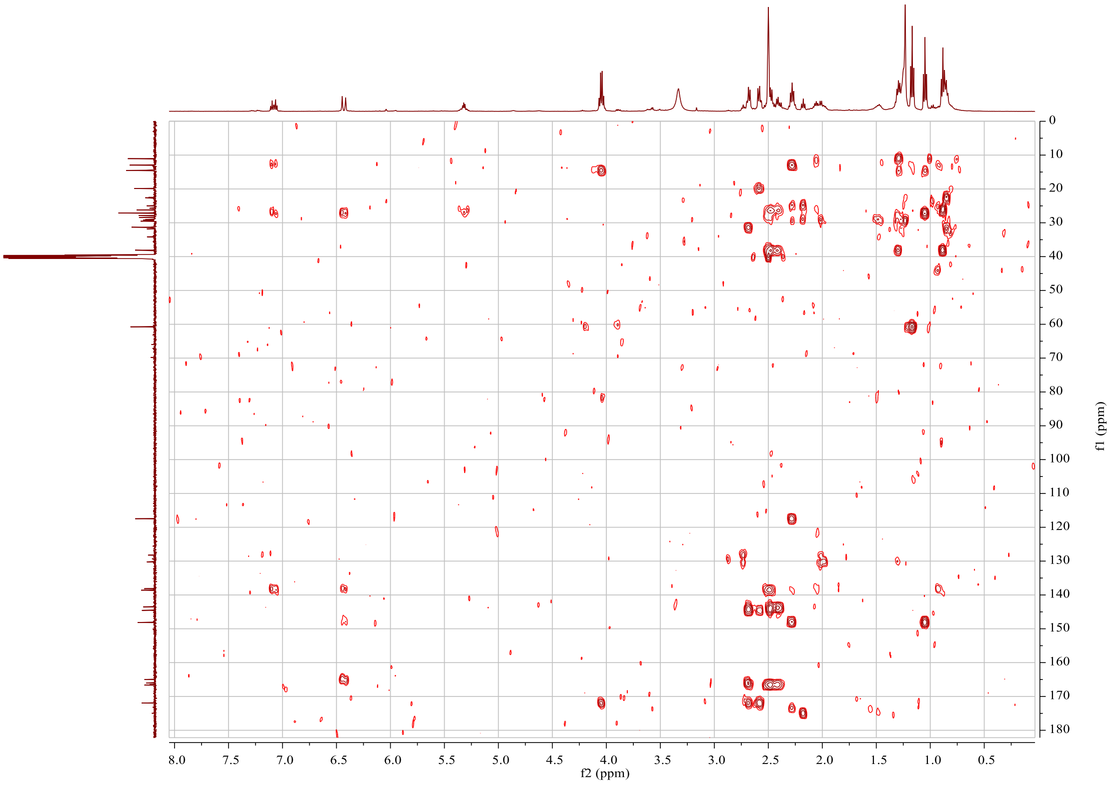

Figure S4. HMBC spectrum of cordyanhydride A ethyl ester (**1**) in DMSO-d_6.

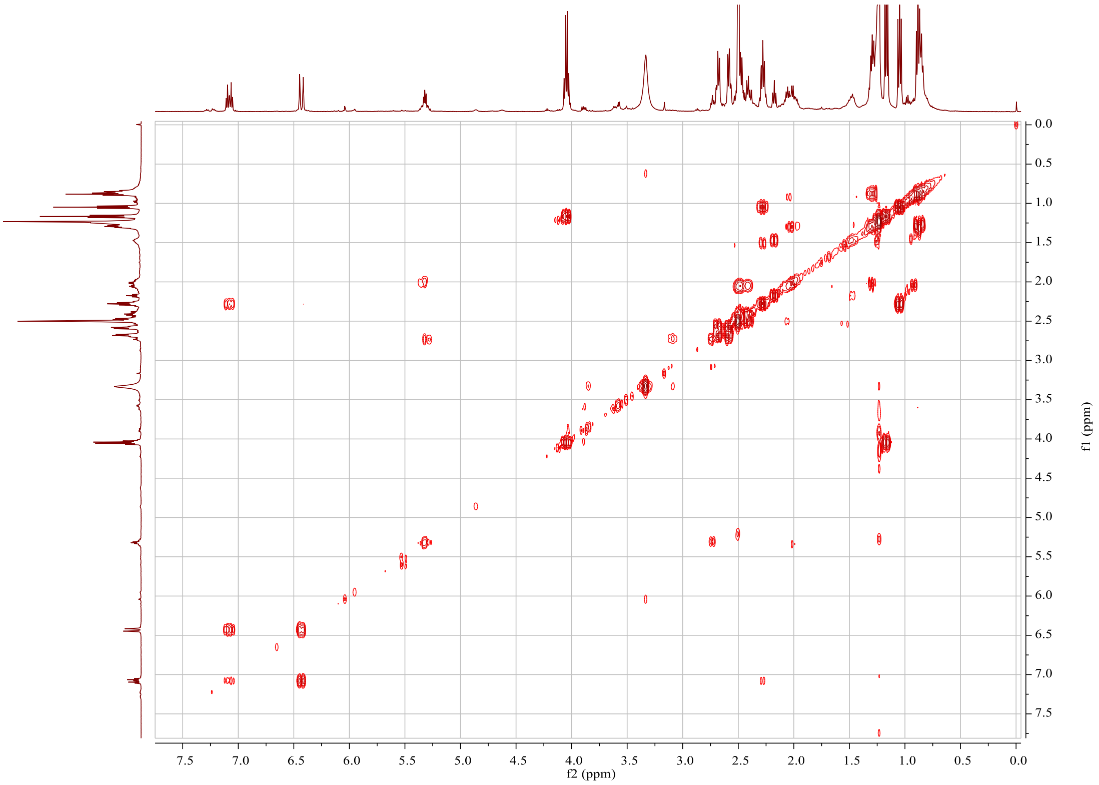

Figure S5. ^{1}H–^{1}H COSY spectrum of cordyanhydride A ethyl ester (**1**) in DMSO-d_6.

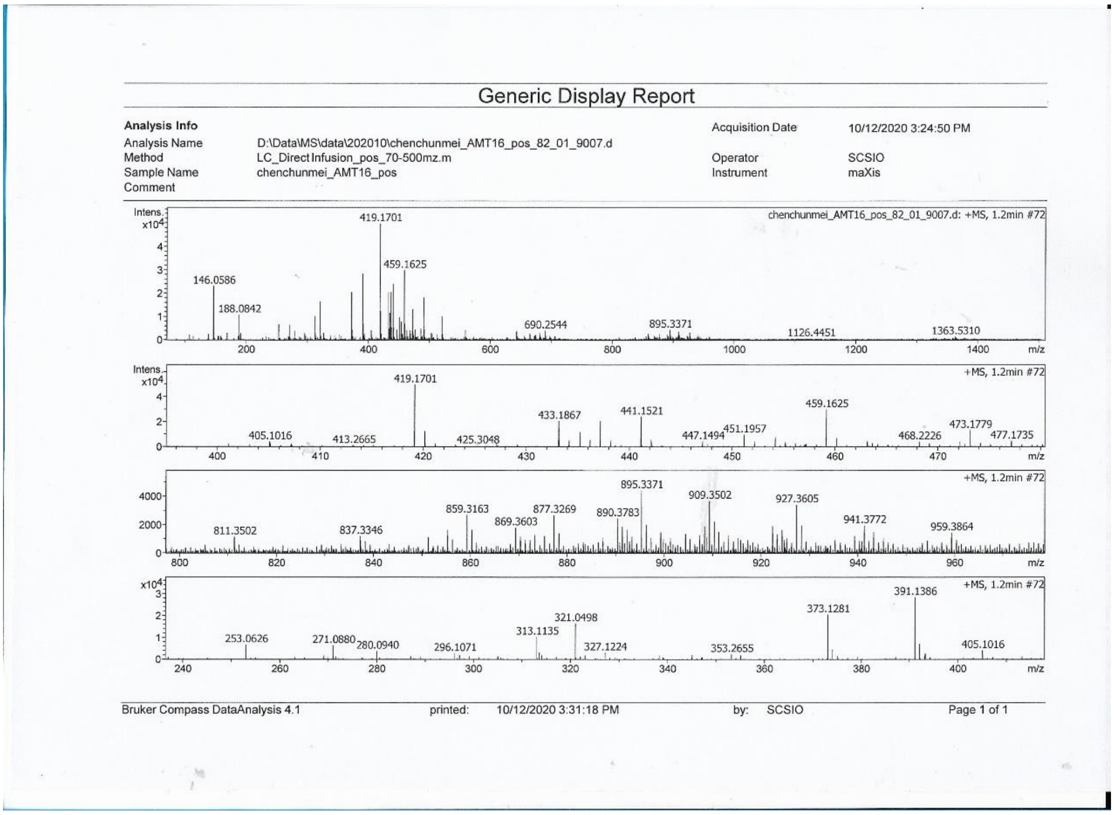

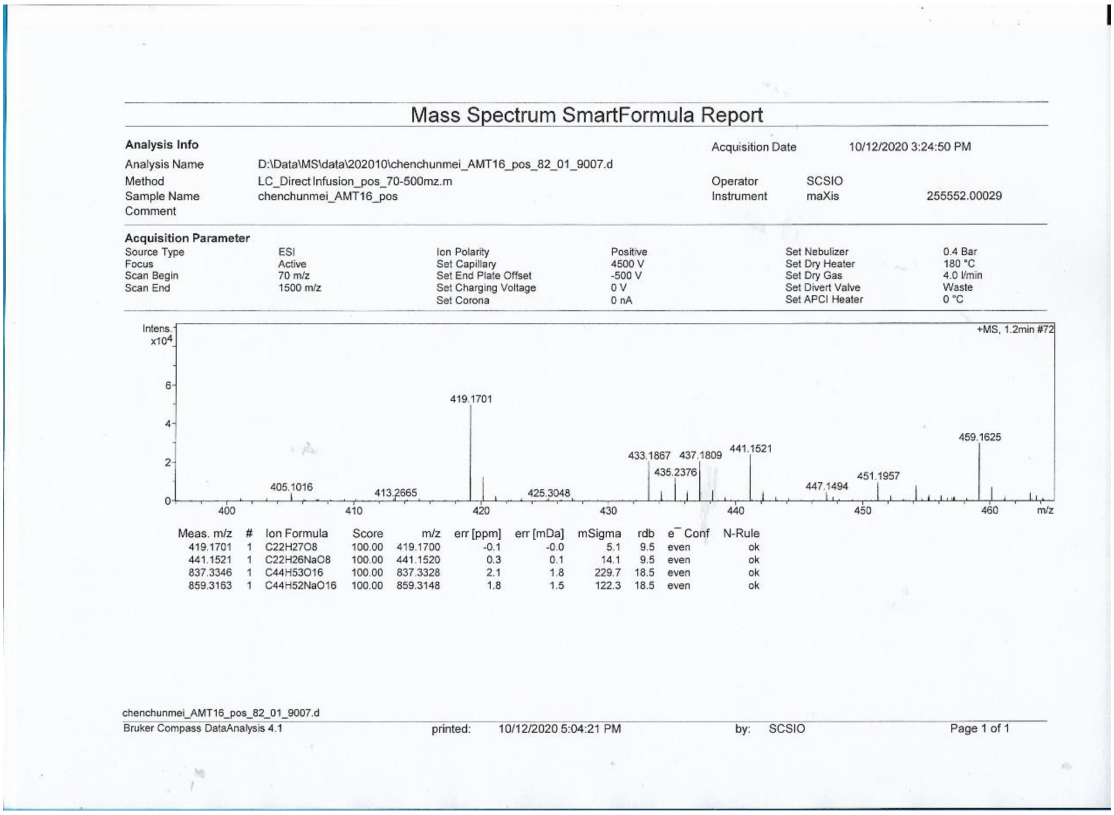

Figure S6. HRESIMS spectrum of cordyanhydride A ethyl ester (**1**).

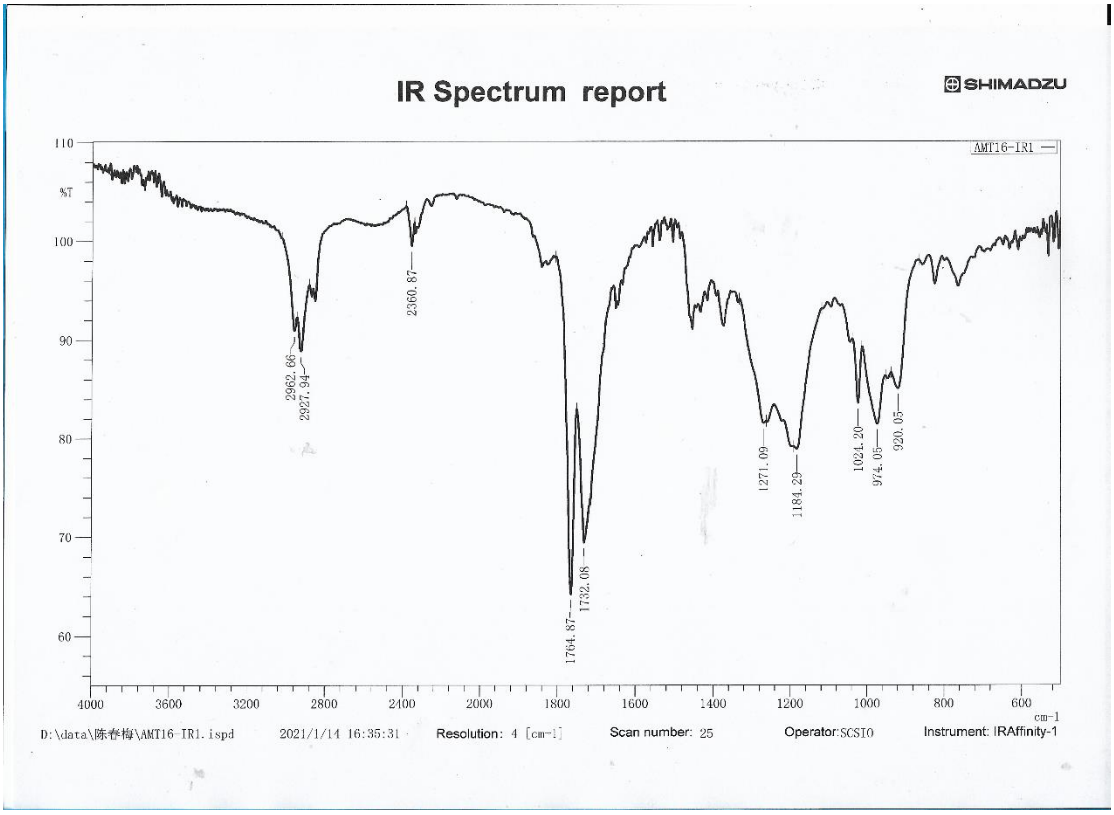

Figure S7. IR spectrum of cordyanhydride A ethyl ester (**1**).

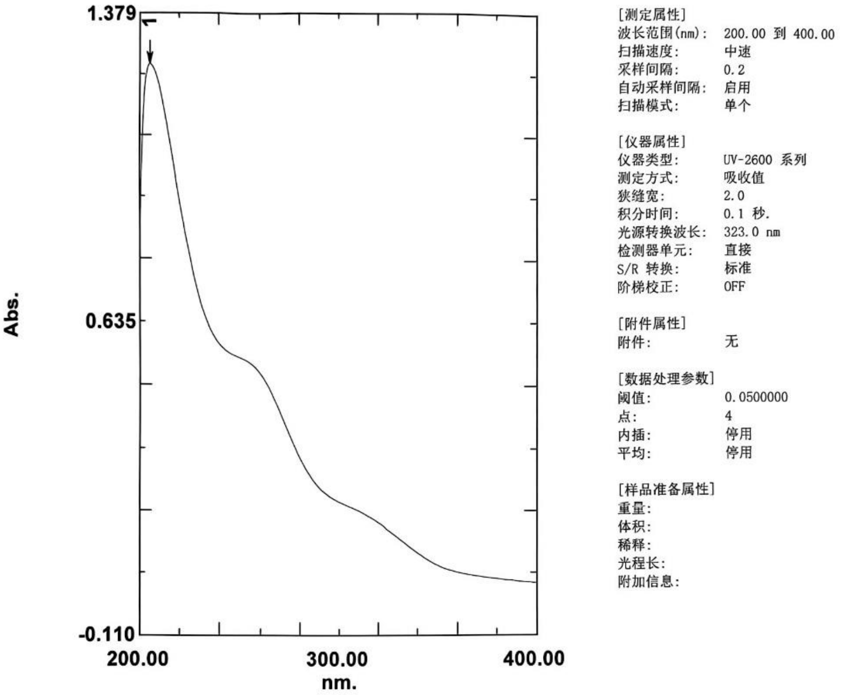

No.	P/V	波长(nm)	Abs.	描述
1	⬆	205.20	1.255	

Figure S8. UV spectrum of cordyanhydride A ethyl ester (**1**) in MeOH.

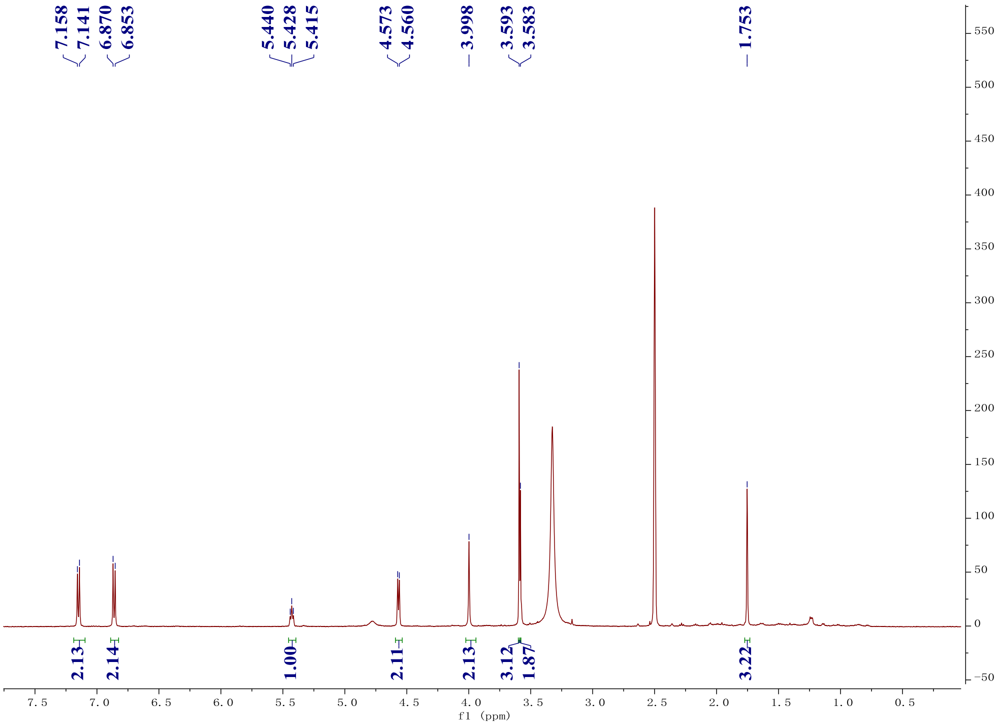

Figure S9. ^{1}H NMR spectrum of stachyline H (**10**) in DMSO-d_6.

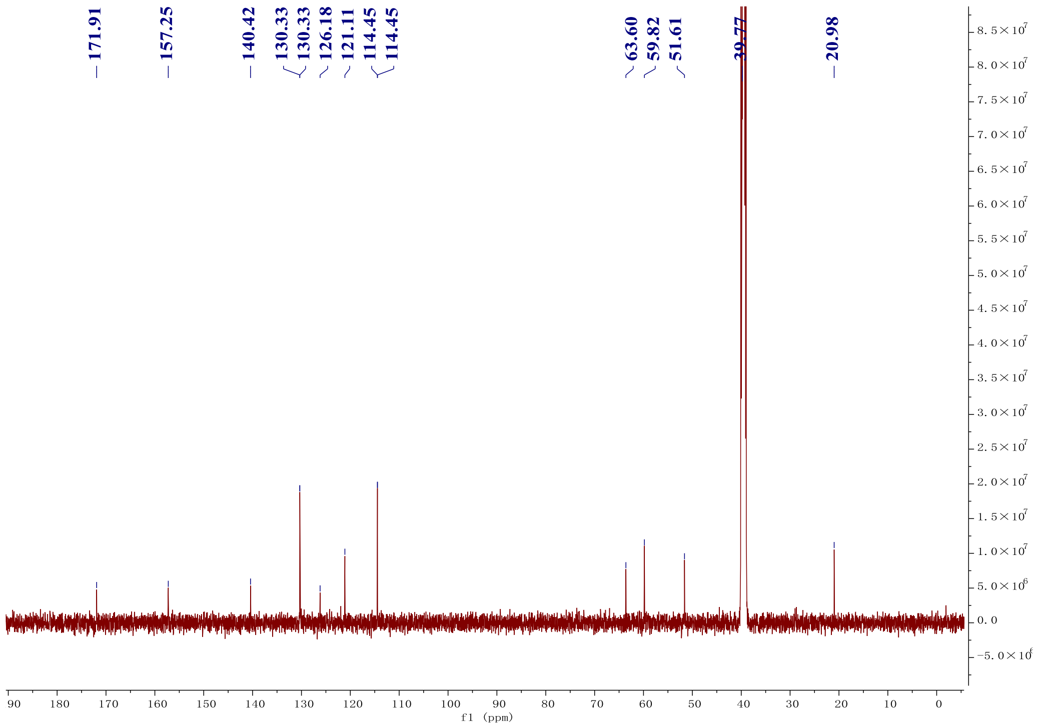

Figure S10. ^{13}C NMR spectrum of stachyline H (**10**) in DMSO-d_6.

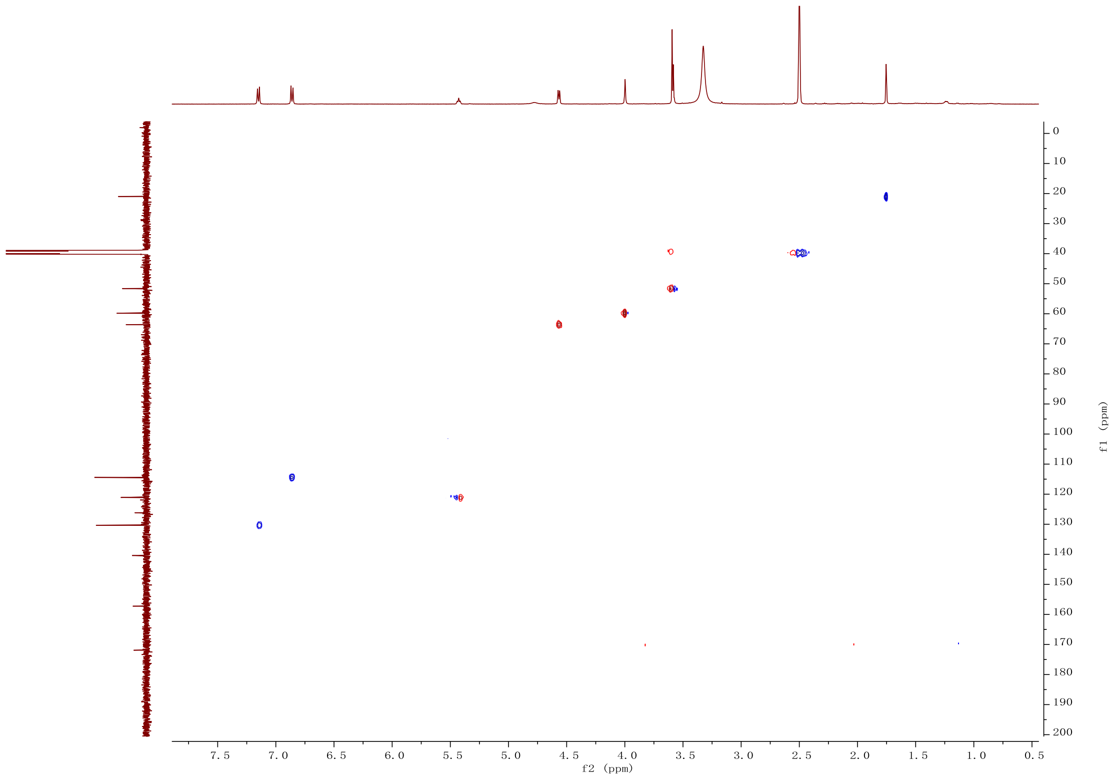

Figure S11. HSQC spectrum of stachyline H (**10**) in DMSO-d_6.

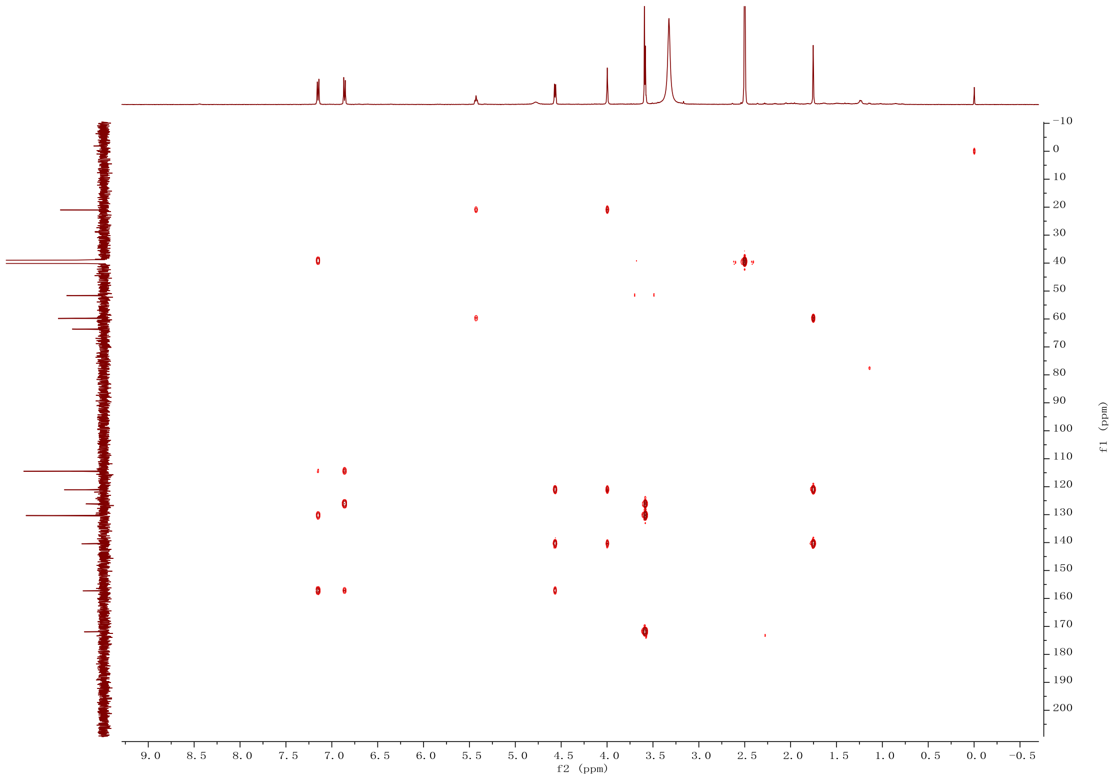

Figure S12. HMBC spectrum of stachyline H (**10**) in DMSO-d_6.

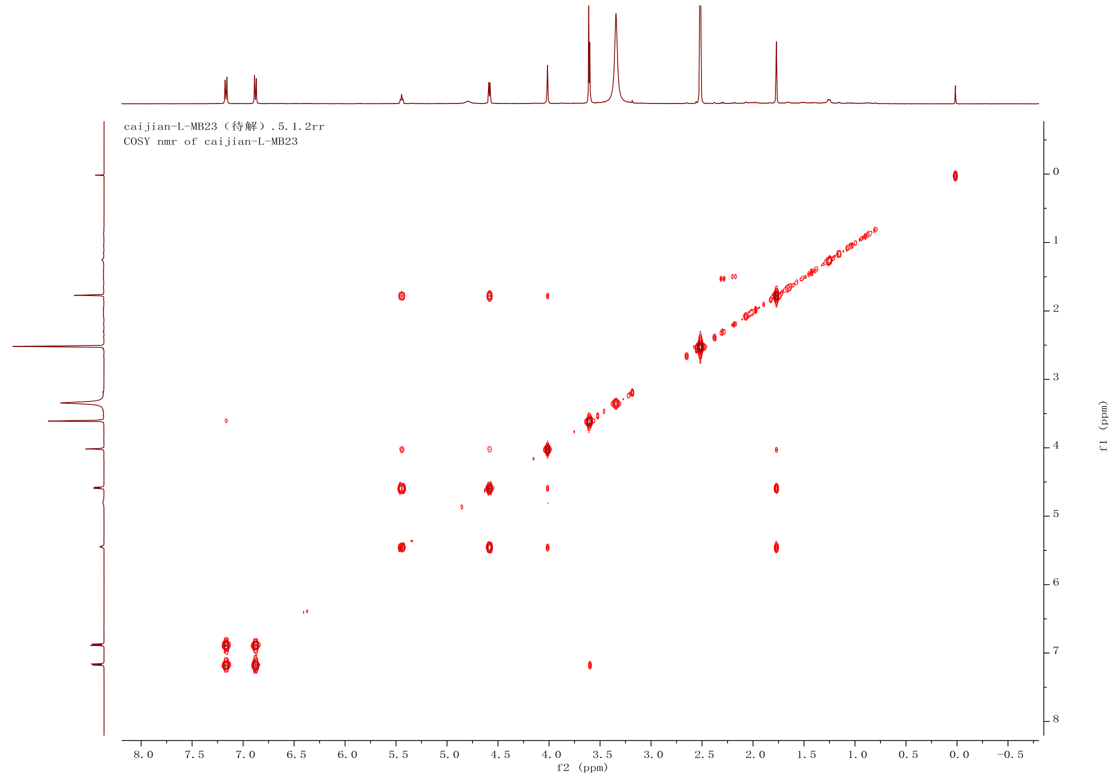

Figure S13. ^{1}H–^{1}H COSY spectrum of stachyline H (**10**) in DMSO-d_6.

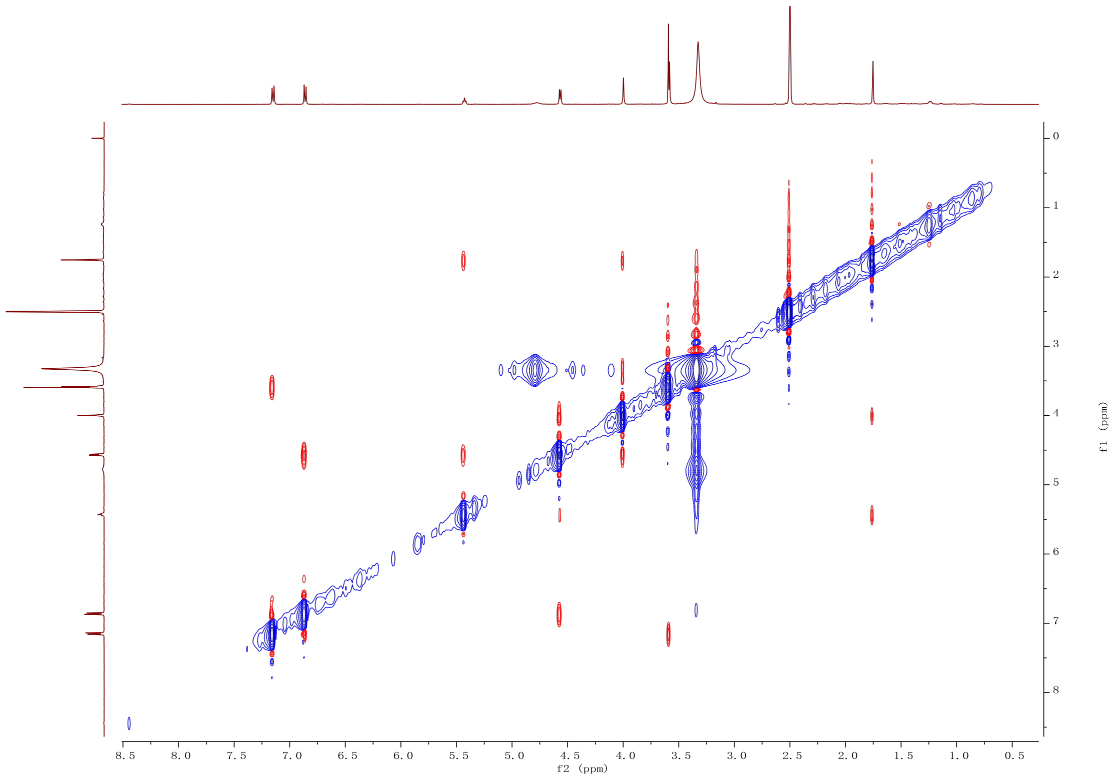

Figure S14. NOESY spectrum of stachyline H (**10**) in DMSO-d_6.

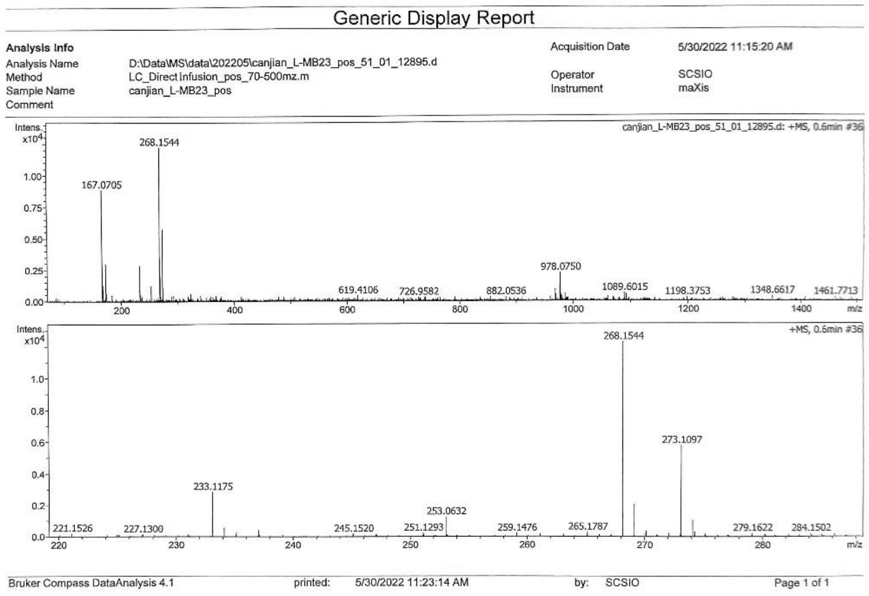

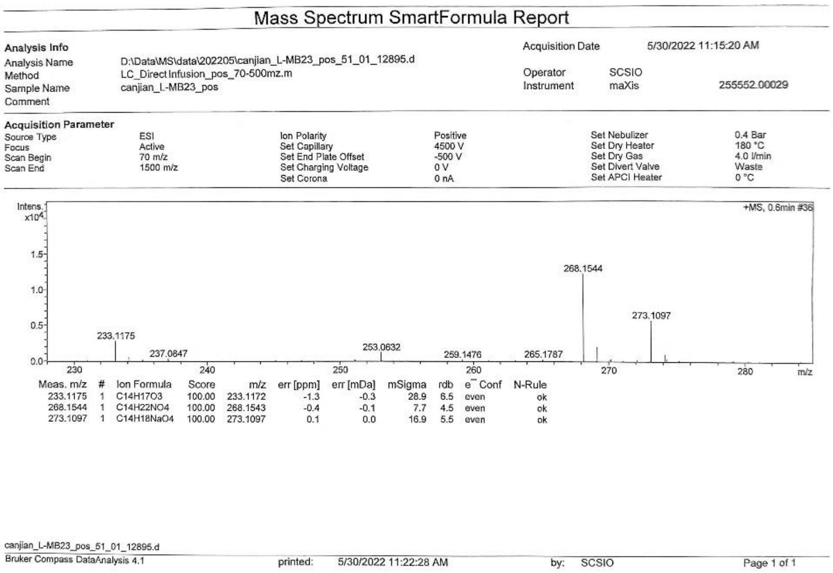

Meas. m/z	#	Ion Formula	Score	m/z	err [ppm]	err [mDa]	mSigma	rdb	e⁻ Conf	N-Rule
233.1175	1	C14H17O3	100.00	233.1172	-1.3	-0.3	28.9	6.5	even	ok
268.1544	1	C14H22NO4	100.00	268.1543	-0.4	-0.1	7.7	4.5	even	ok
273.1097	1	C14H18NaO4	100.00	273.1097	0.1	0.0	16.9	5.5	even	ok

Figure S15. HRESIMS spectrum of stachyline H (**10**).

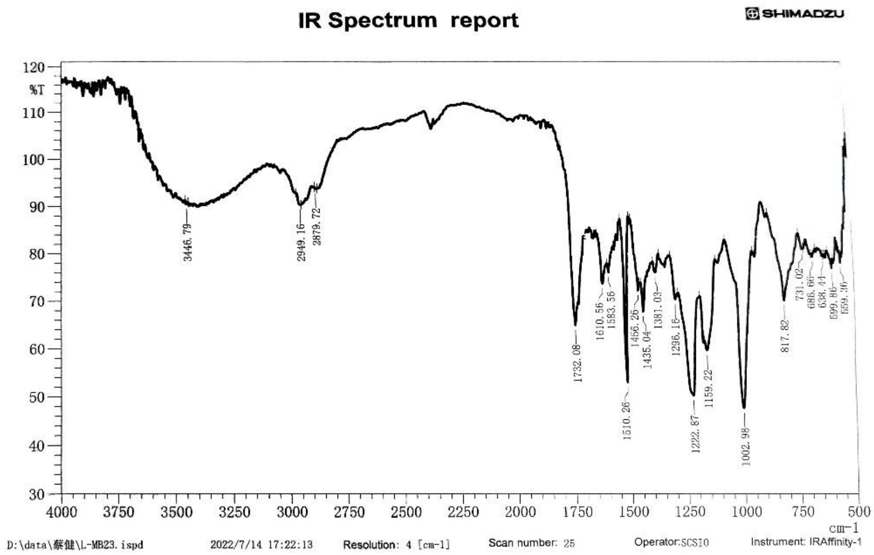

Figure S16. IR spectrum of stachyline H (**10**).

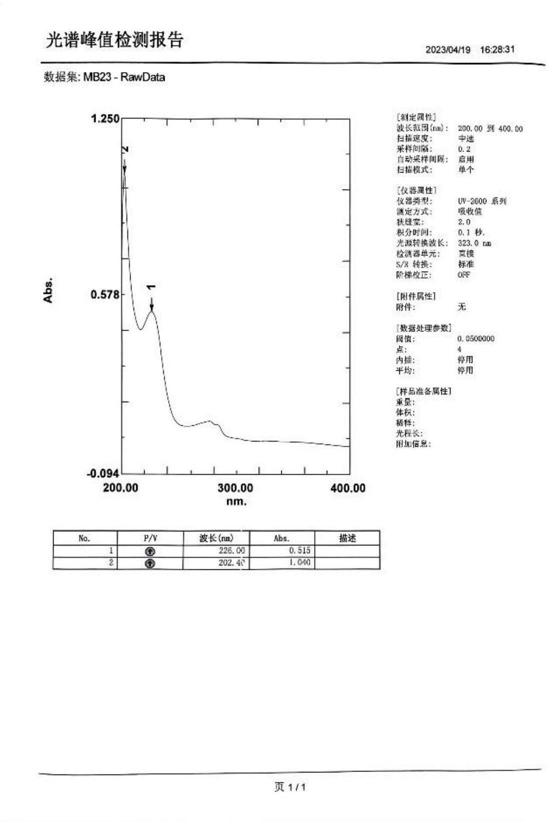

Figure S17. UV spectrum of stachyline H (**10**) in MeOH.

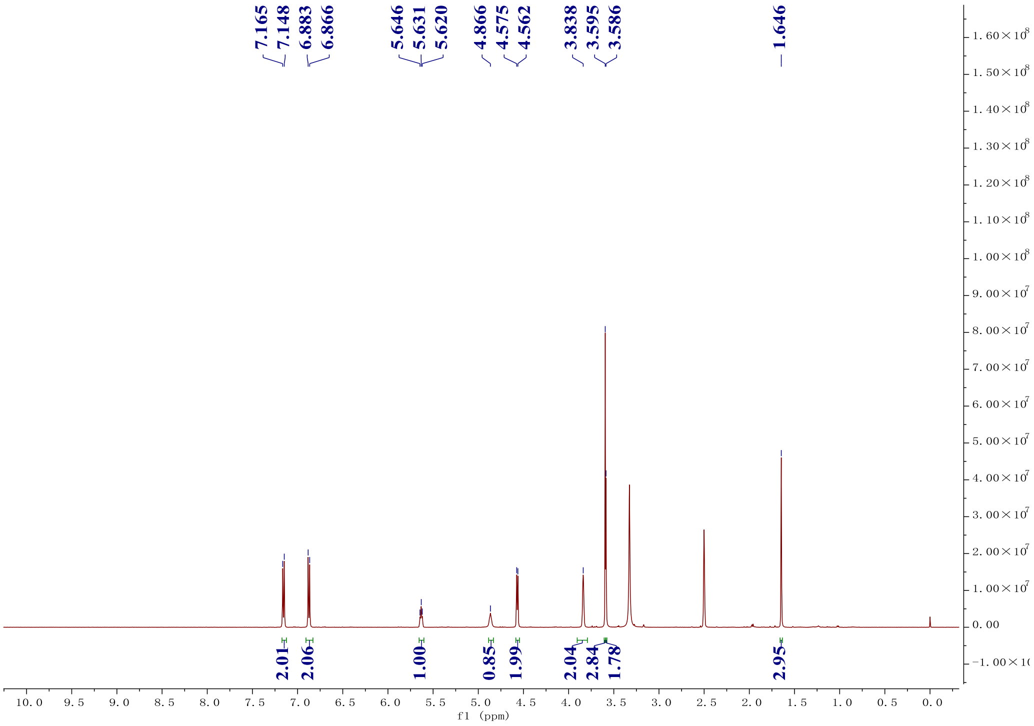

Figure S18. ^{1}H NMR spectrum of stachyline I (**11**) in DMSO-d_6.

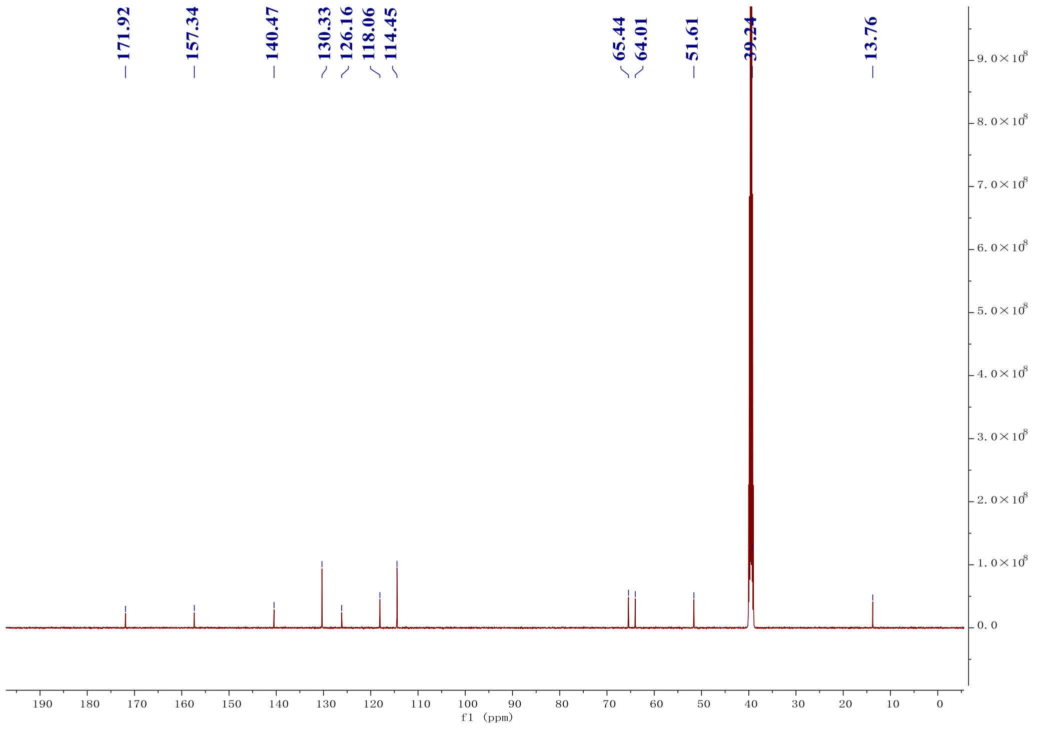

Figure S19. ^{13}C NMR spectrum of stachyline I (**11**) in DMSO-d_6.

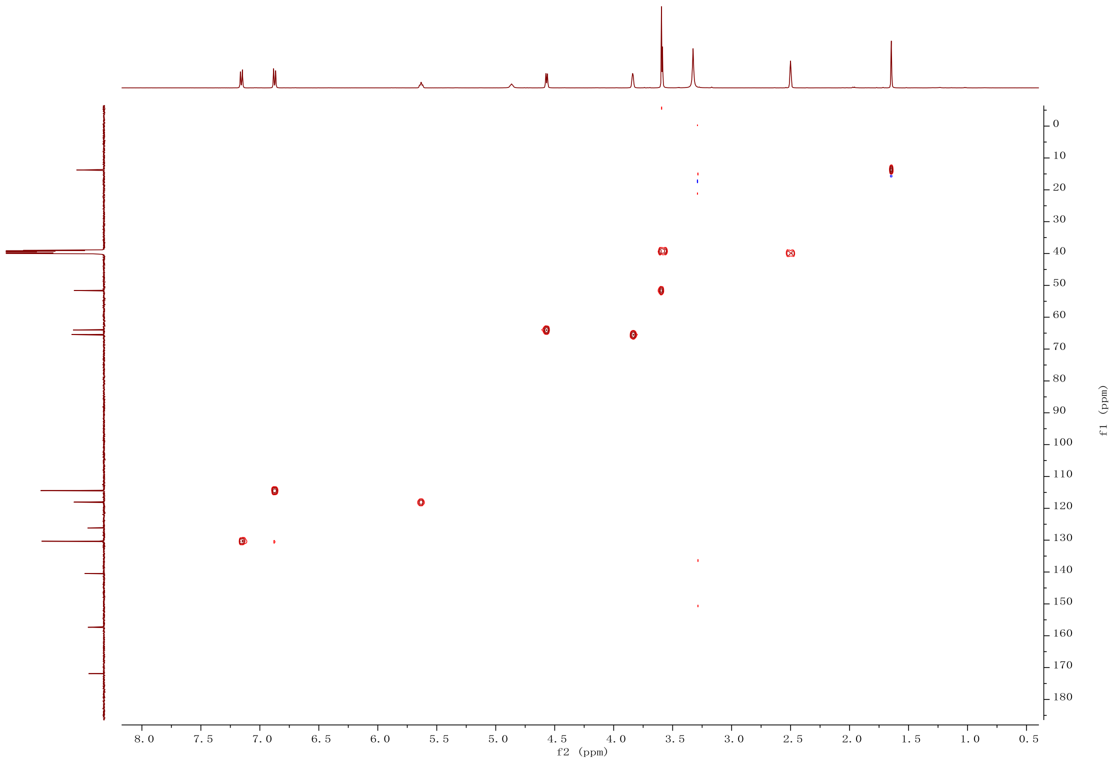

Figure S20. HSQC spectrum of stachyline I (**11**) in DMSO-d_6.

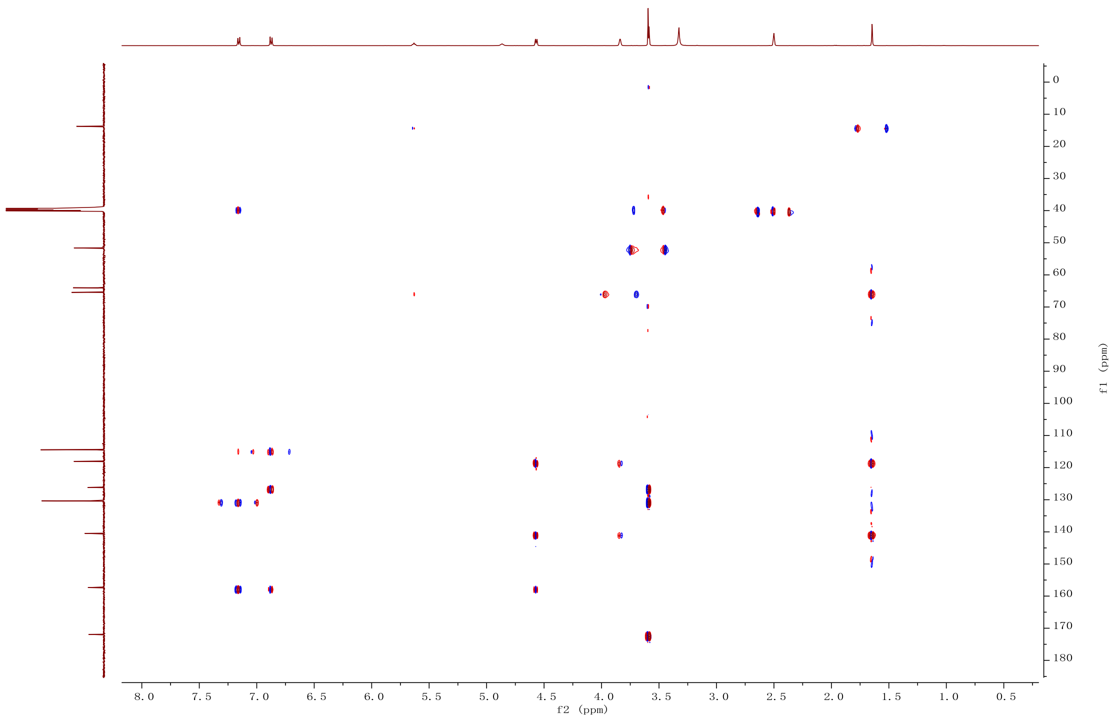

Figure S21. HMBC spectrum of stachyline I (**11**) in DMSO-d_6.

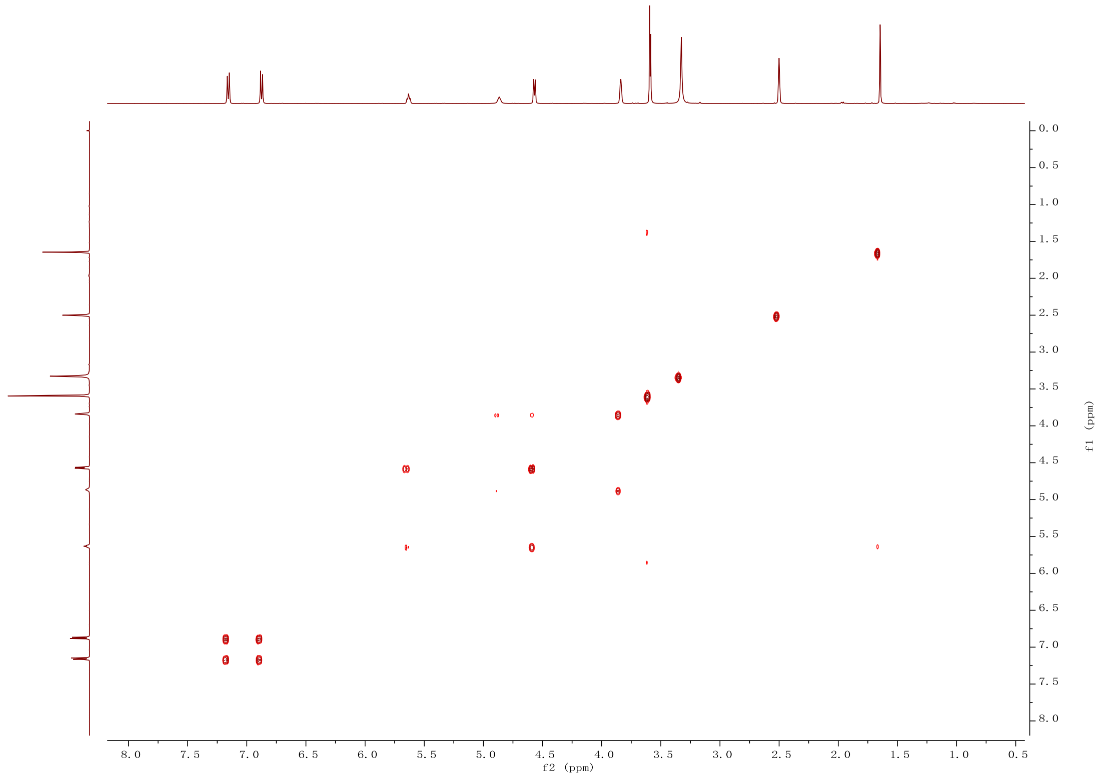

Figure S22. ^{1}H–^{1}H COSY spectrum of stachyline I (**11**) in DMSO-d_6.

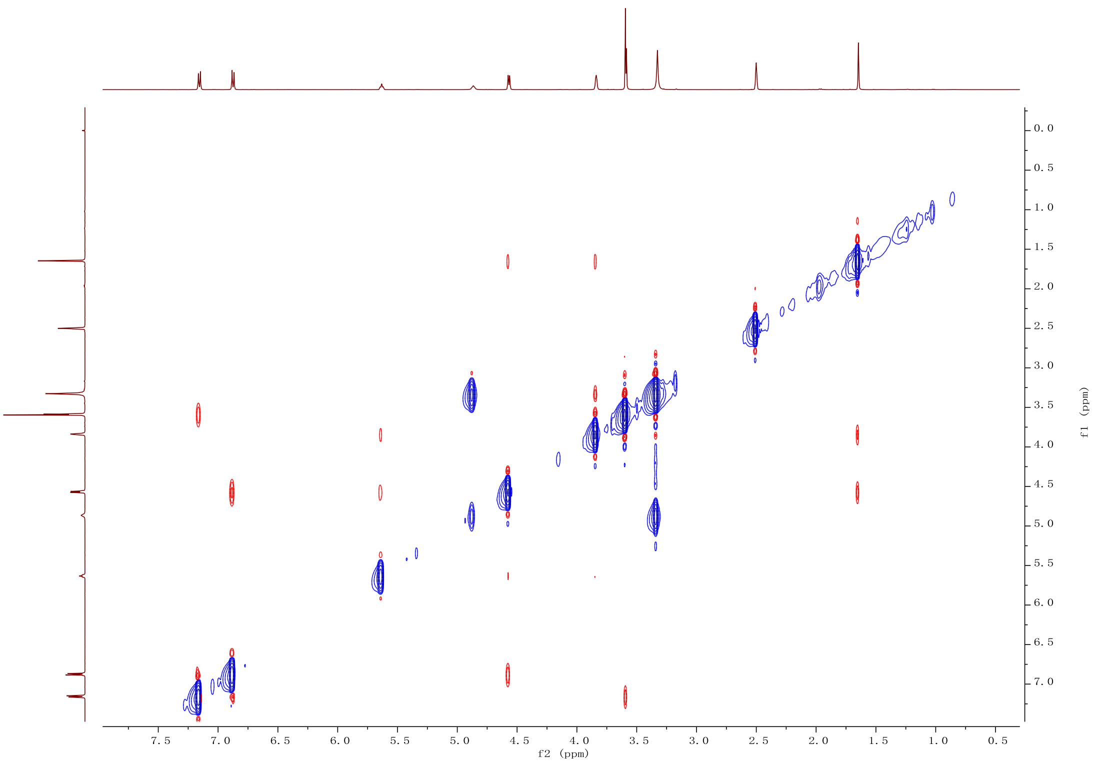

Figure S23. NOESY spectrum of stachyline I (**11**) in DMSO-d_6.

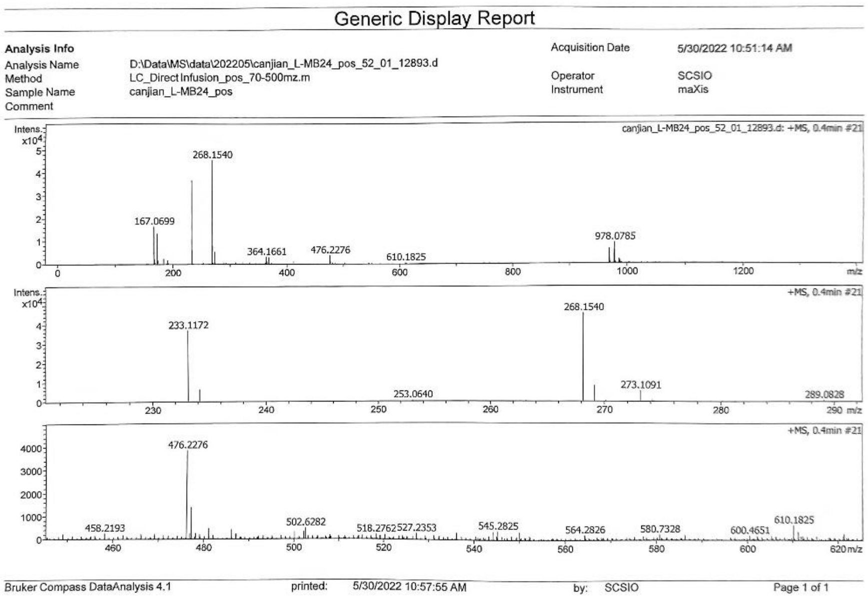

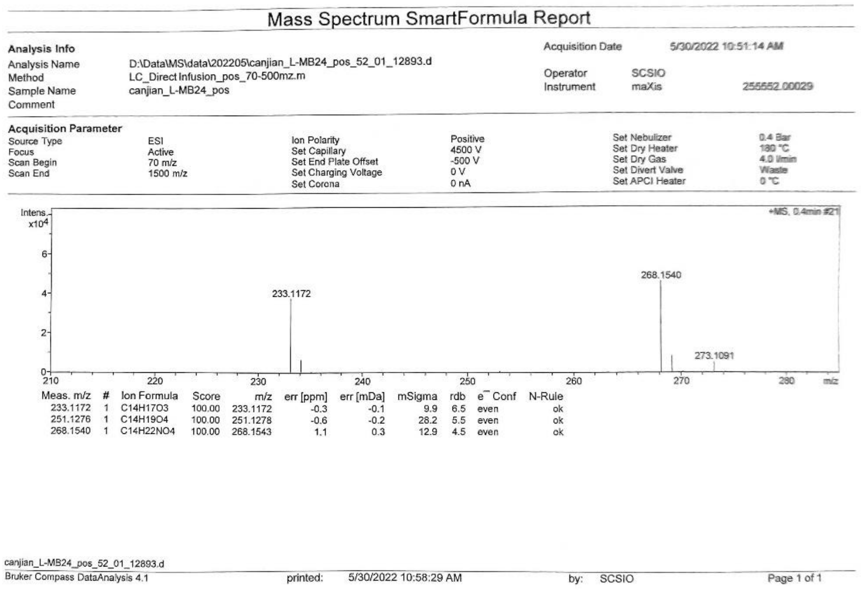

Figure S24. HRESIMS spectrum of stachyline I (**11**).

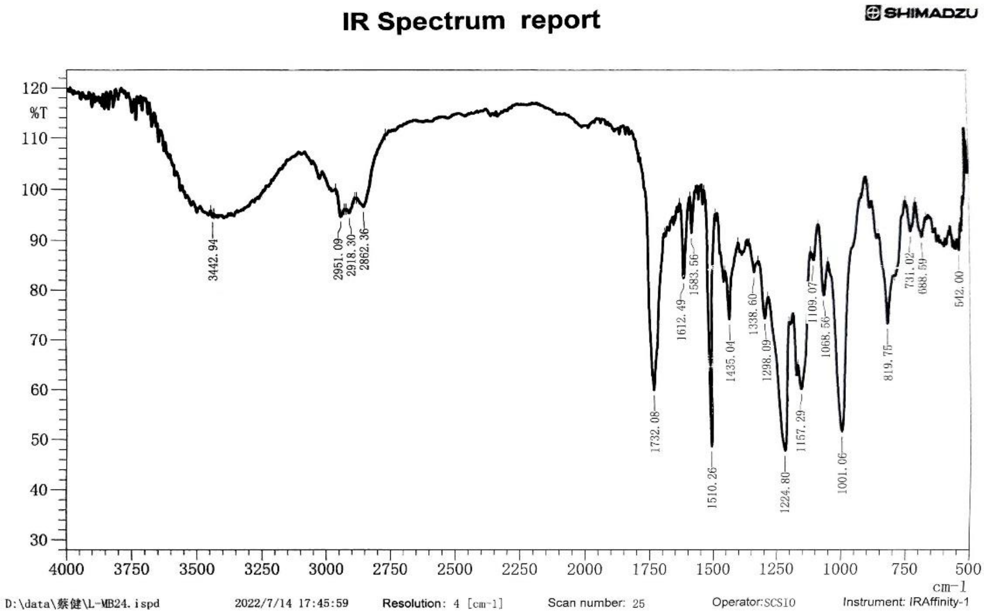

Figure S25. IR spectrum of stachyline I (**11**).

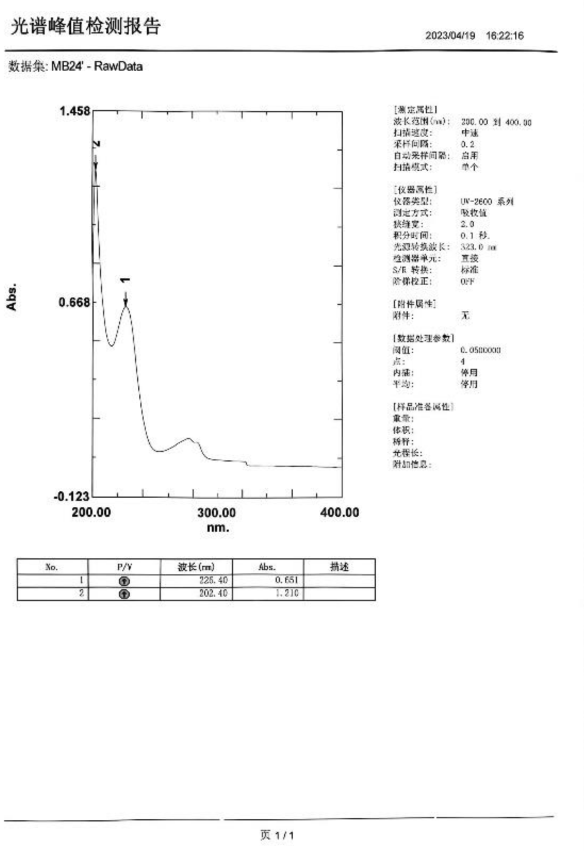

Figure S26. UV spectrum of stachyline I (**11**) in MeOH.

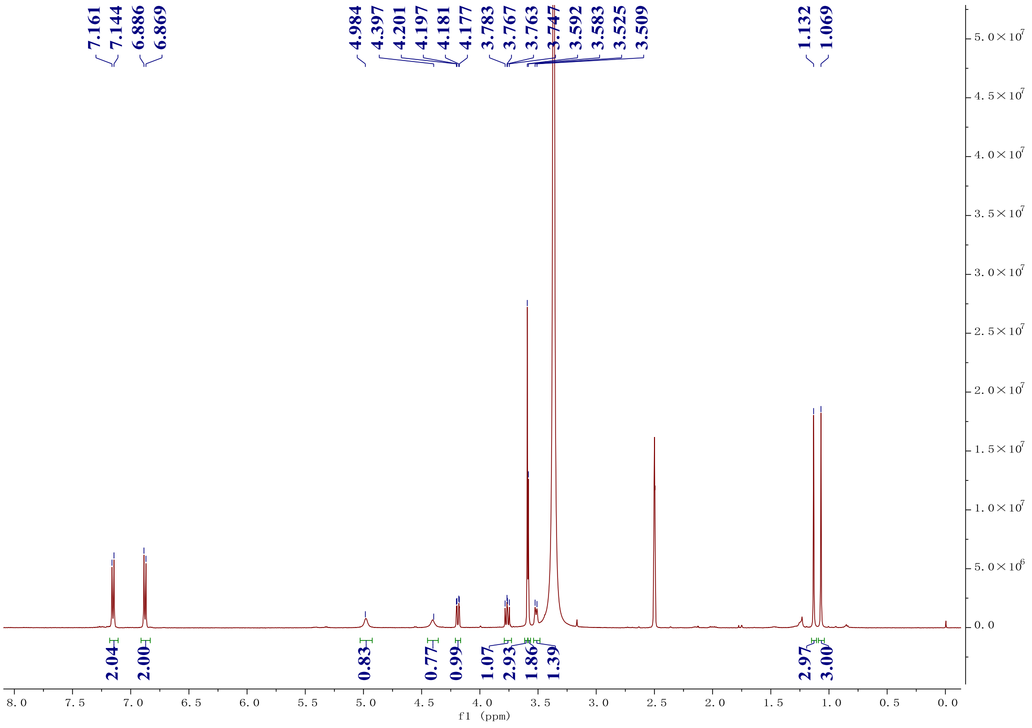

Figure S27. ^{1}H NMR spectrum of stachyline J (**12**) in DMSO-d_6.

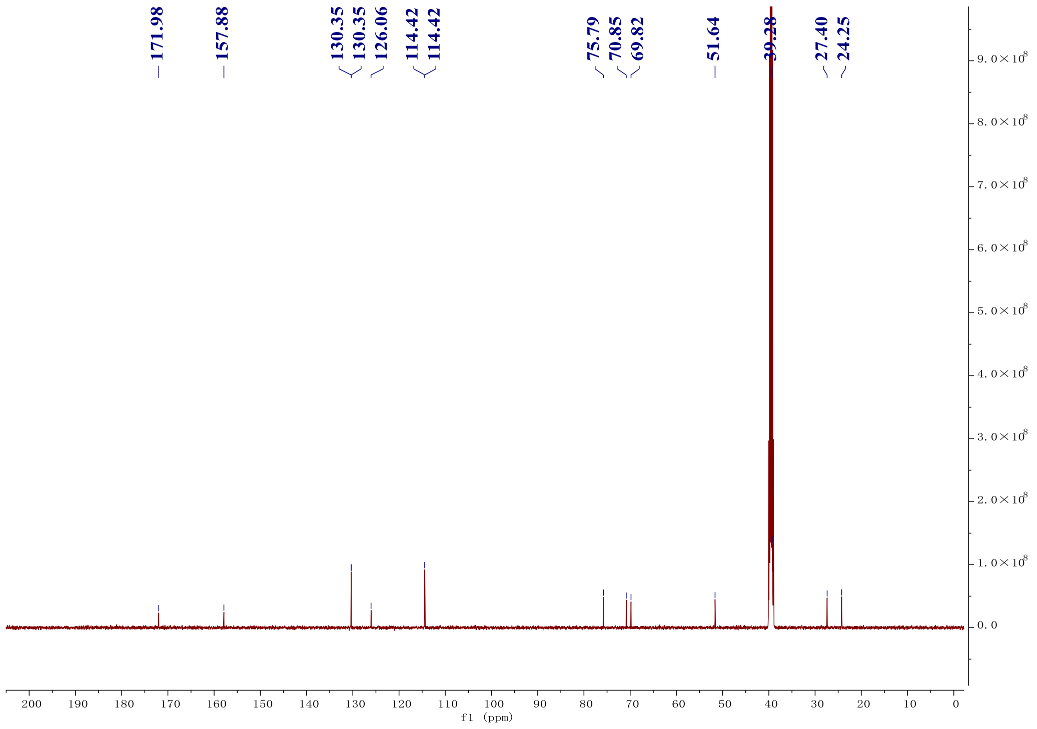

Figure S28. ^{13}C NMR spectrum of stachyline J (**12**) in DMSO-d_6.

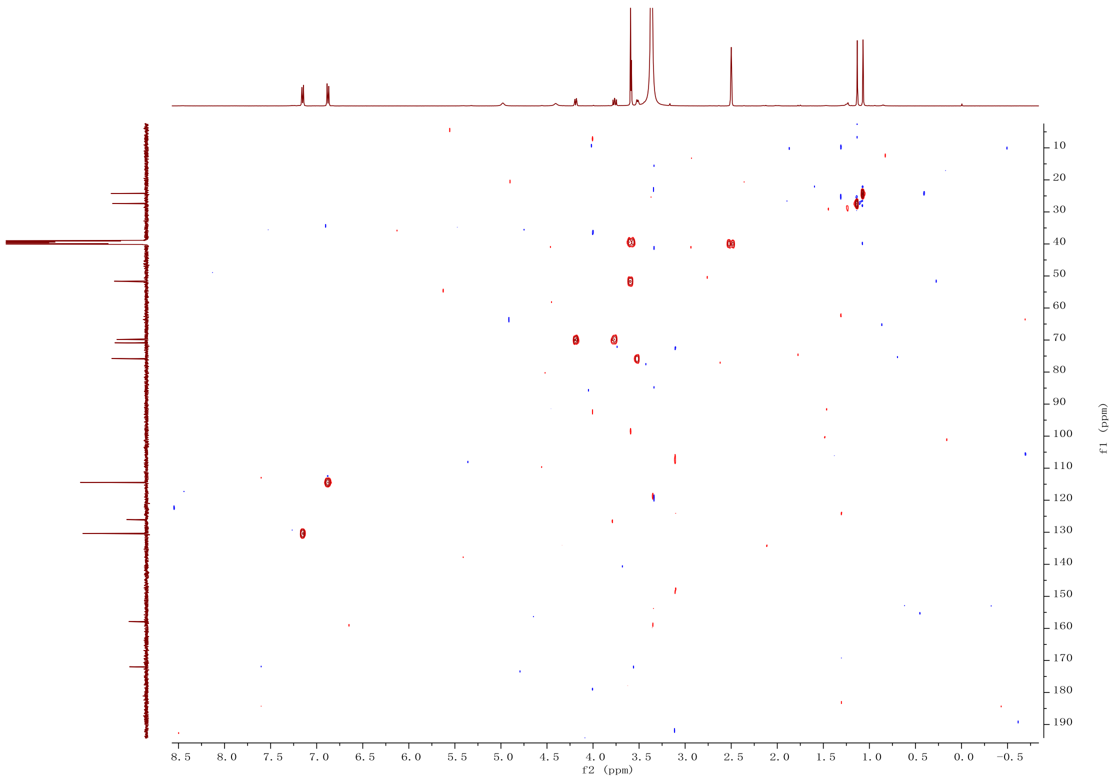

Figure S29. HSQC spectrum of stachyline J (**12**) in DMSO-d_6.

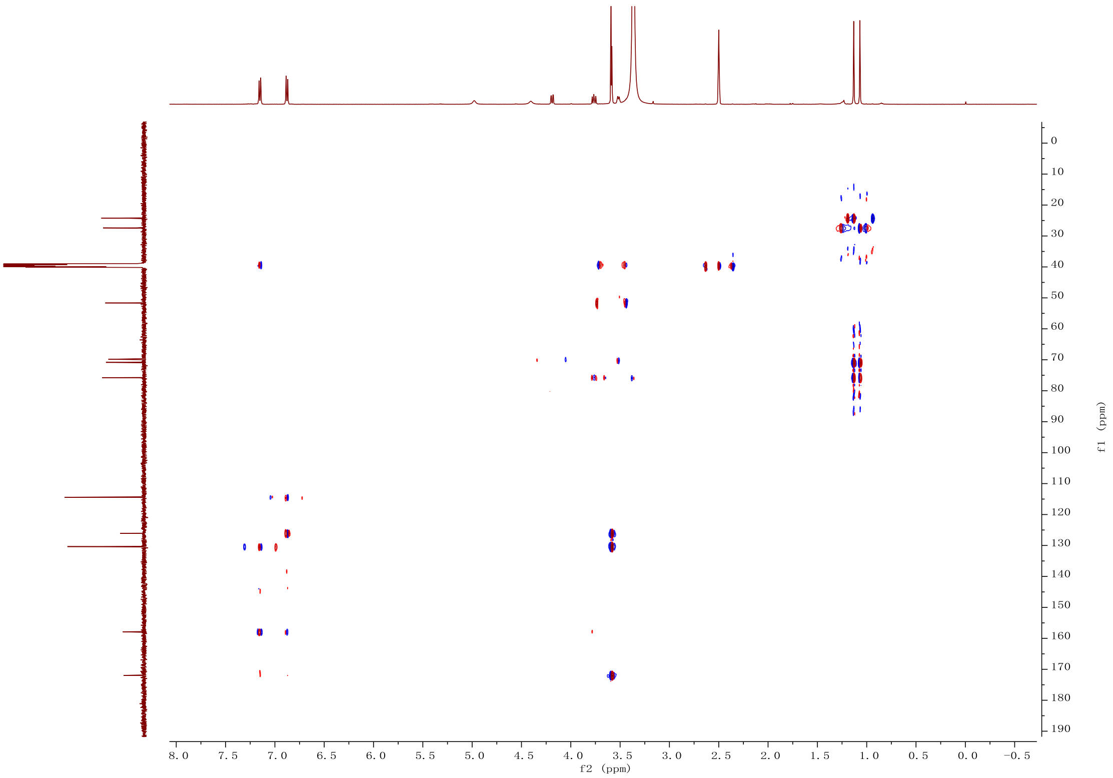

Figure S30. HMBC spectrum of stachyline J (**12**) in DMSO-d_6.

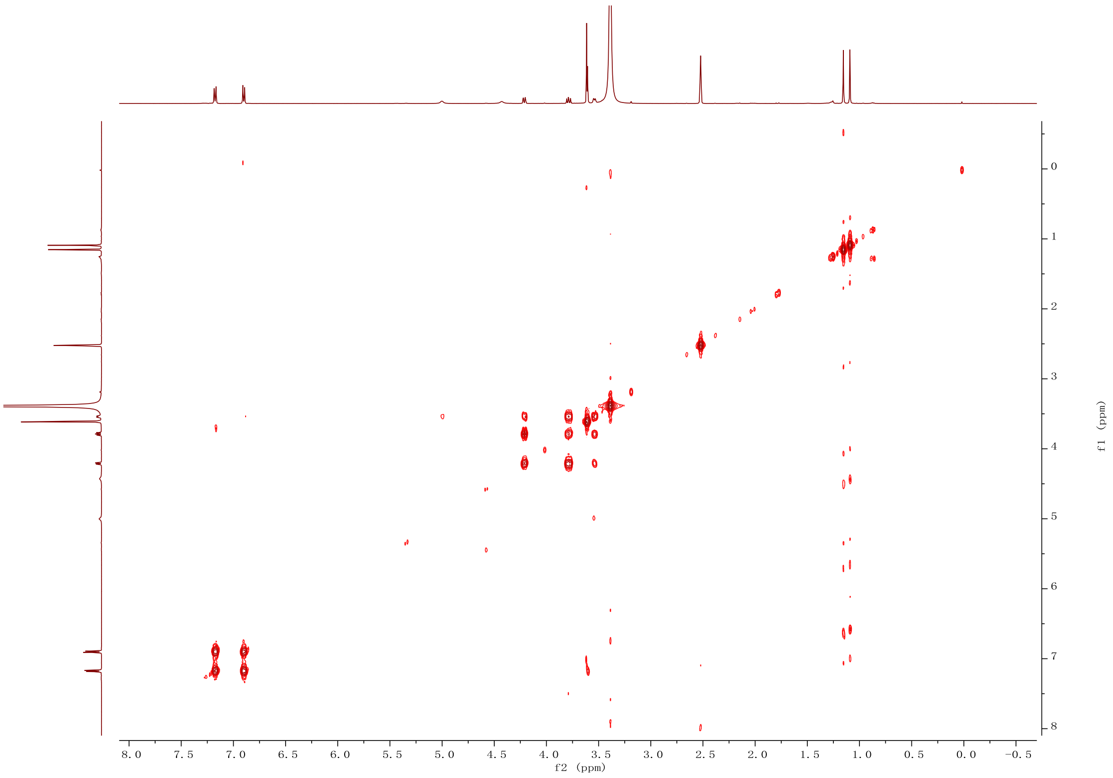

Figure S31. ^{1}H–^{1}H COSY spectrum of stachyline J (**12**) in DMSO-d_6.

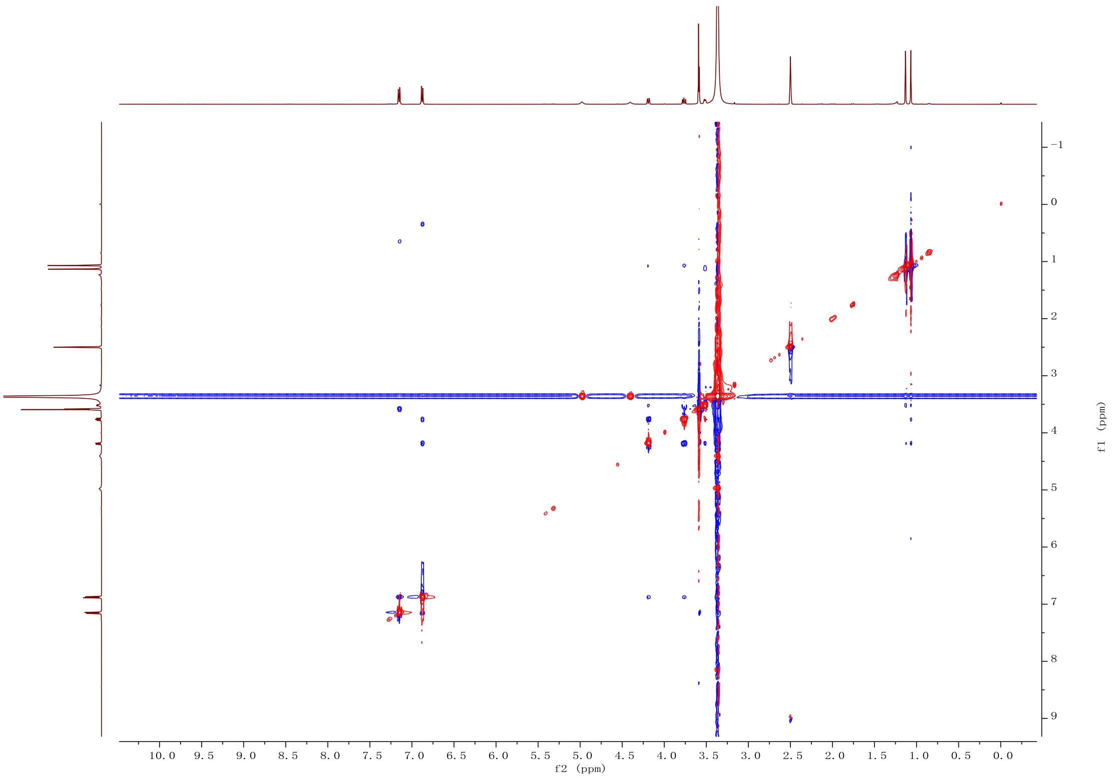

Figure S32. NOESY spectrum of stachyline J (**12**) in DMSO-d6.

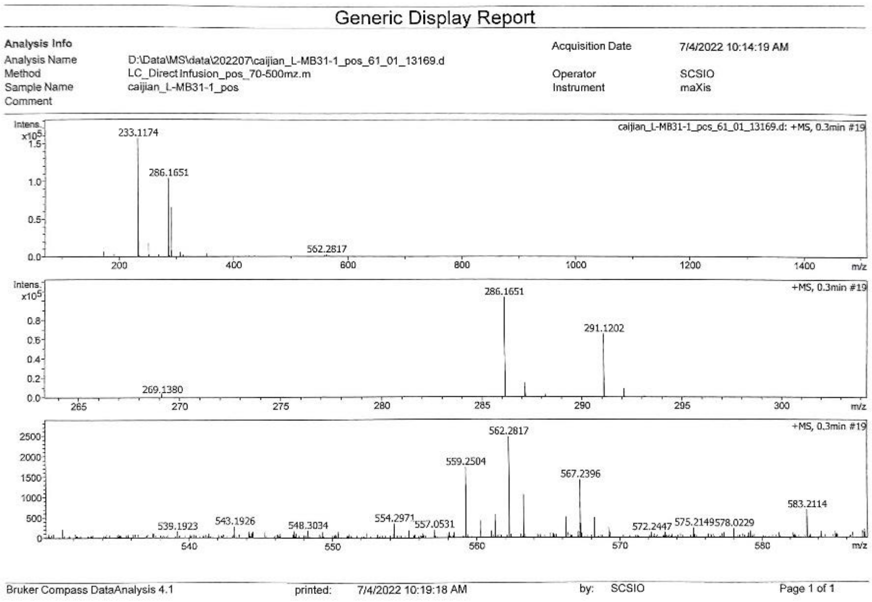

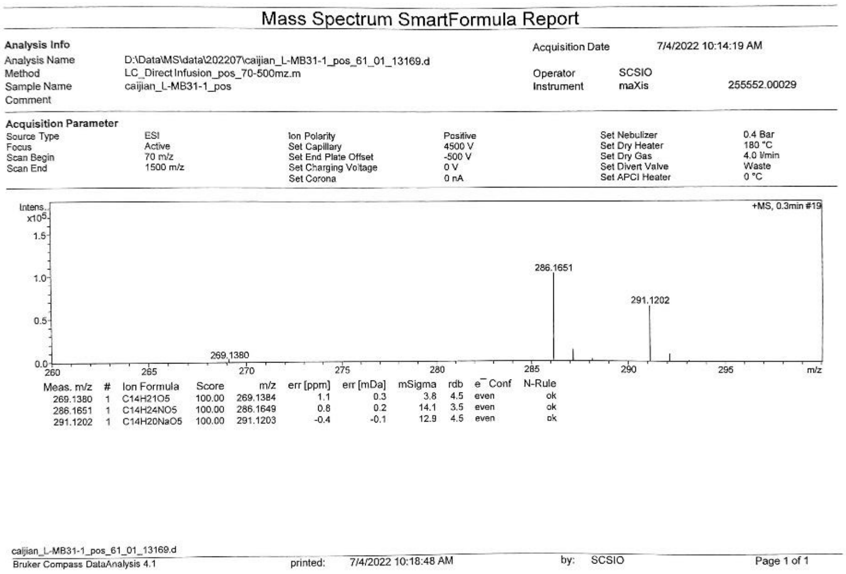

Meas. m/z	#	Ion Formula	Score	m/z	err [ppm]	err [mDa]	mSigma	rdb	e⁻ Conf	N-Rule
269.1380	1	C14H21O5	100.00	269.1384	1.1	0.3	3.8	4.5	even	ok
286.1651	1	C14H24NO5	100.00	286.1649	0.8	0.2	14.1	3.5	even	ok
291.1202	1	C14H20NaO5	100.00	291.1203	-0.4	-0.1	12.9	4.5	even	ok

caijian_L-MB31-1_pos_61_01_13169.d
Bruker Compass DataAnalysis 4.1 printed: 7/4/2022 10:18:48 AM by: SCSIO Page 1 of 1

Figure S33. HRESIMS spectrum of stachyline J (**12**).

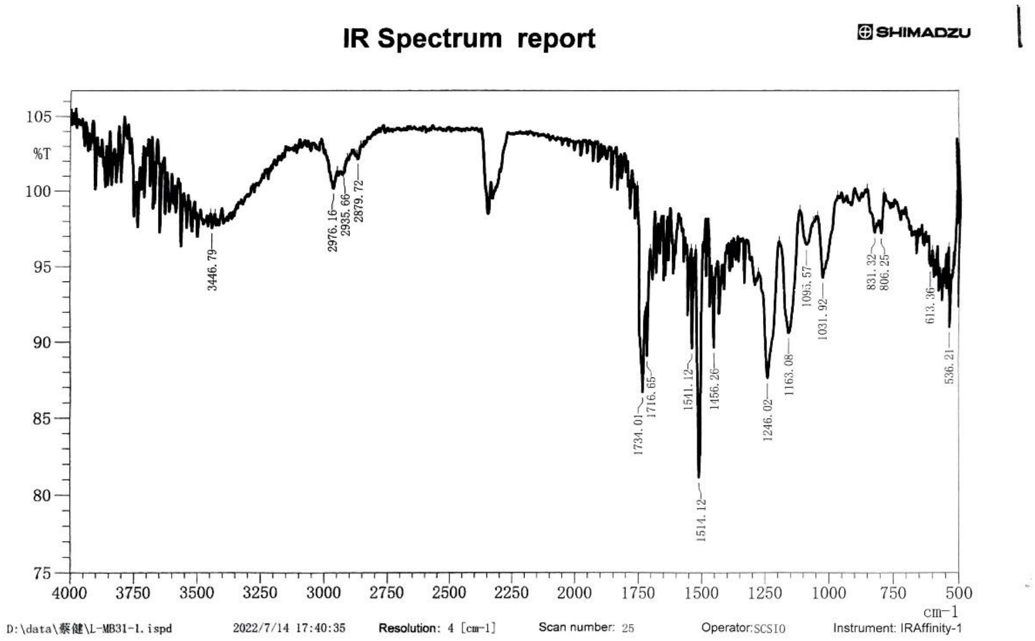

Figure S34. IR spectrum of stachyline J (**12**).

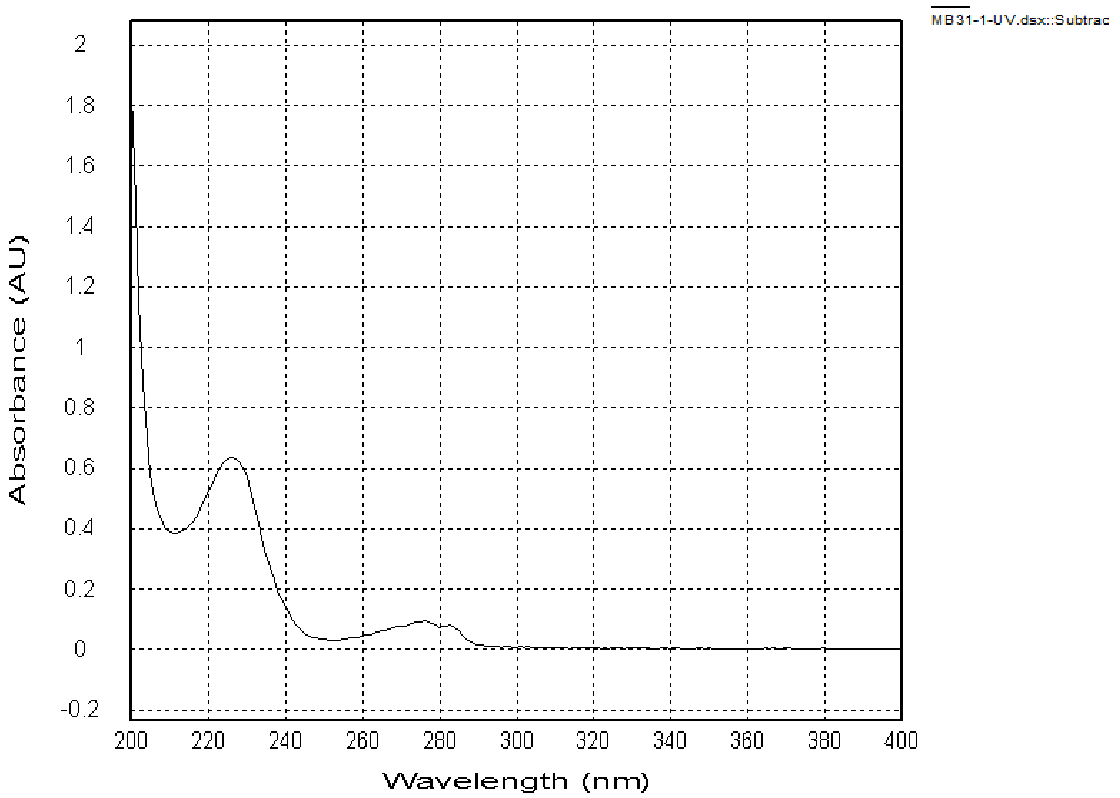

Figure S35. UV spectrum of stachyline J (**12**) in MeOH.

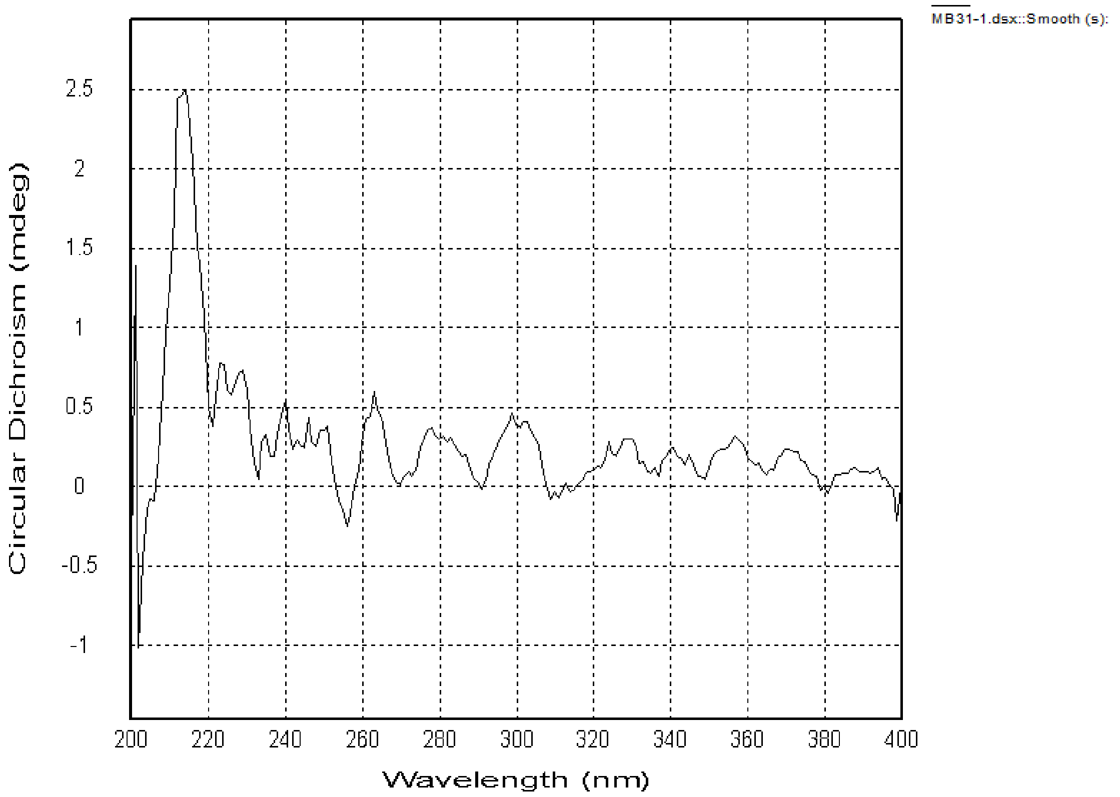

Figure S36. ECD spectrum of stachyline J (**12**) in MeOH.

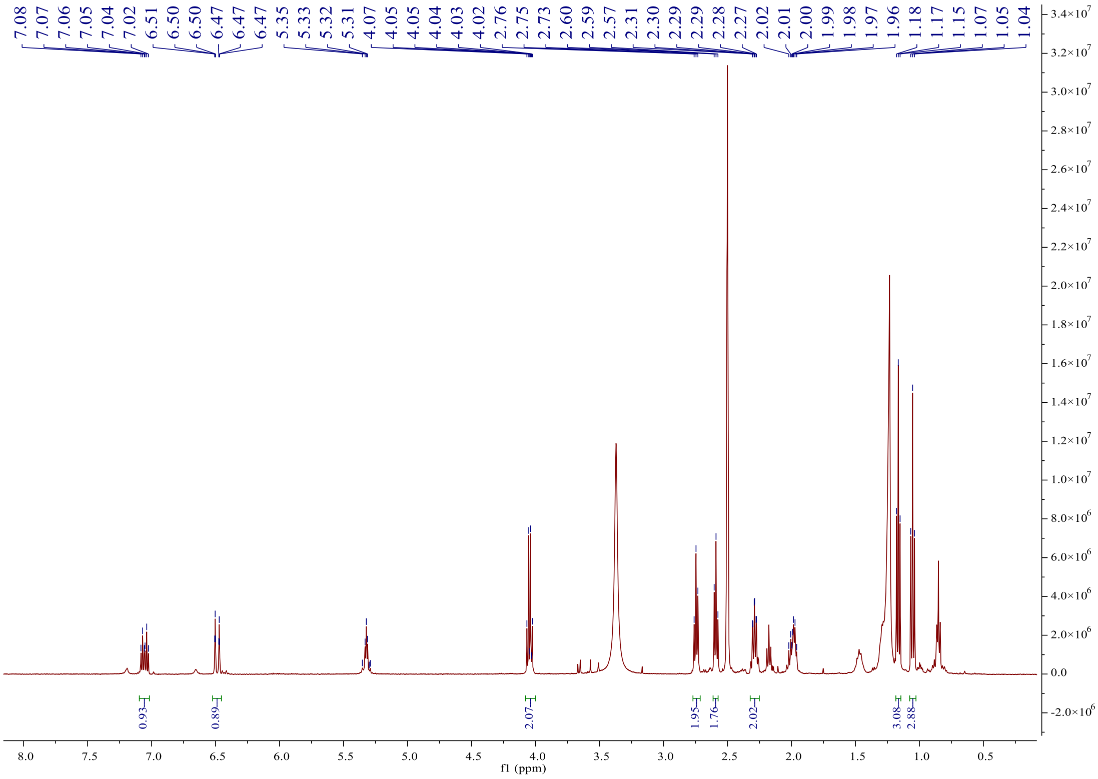

Figure S37. ^{1}H NMR spectrum of maleicanhydridane (**4**) in DMSO-d_6.

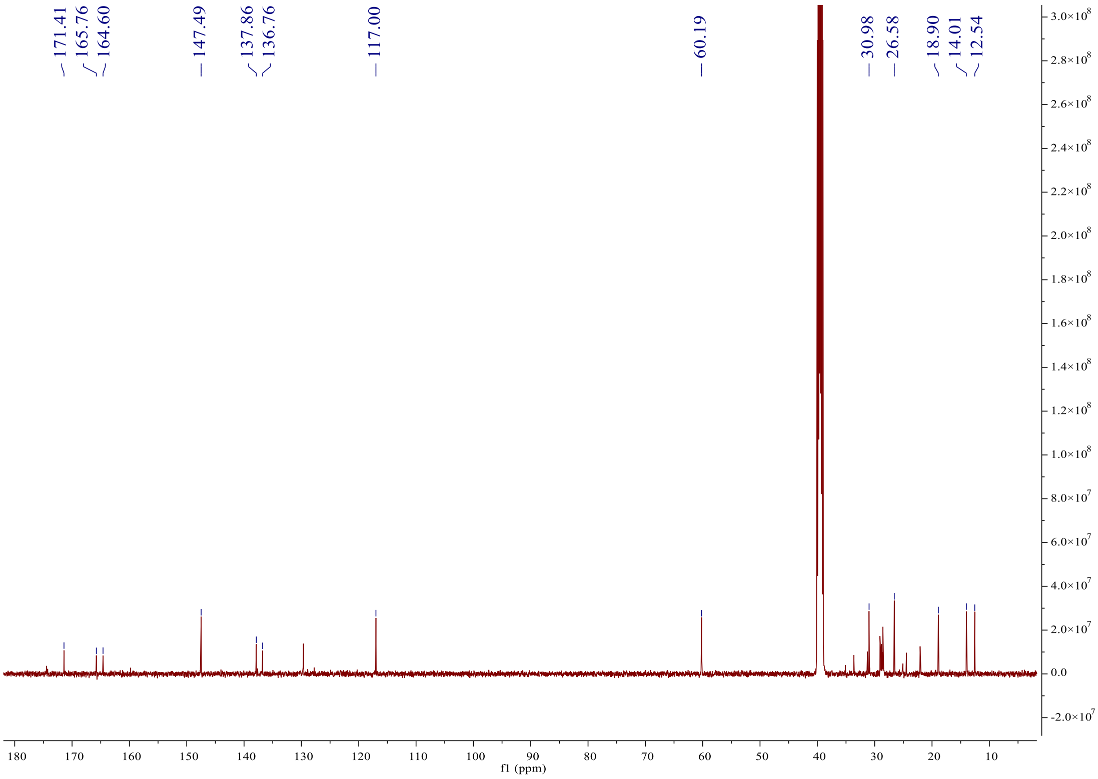

Figure S38. 13C NMR spectrum of maleicanhydridane (**4**) in DMSO-d6.

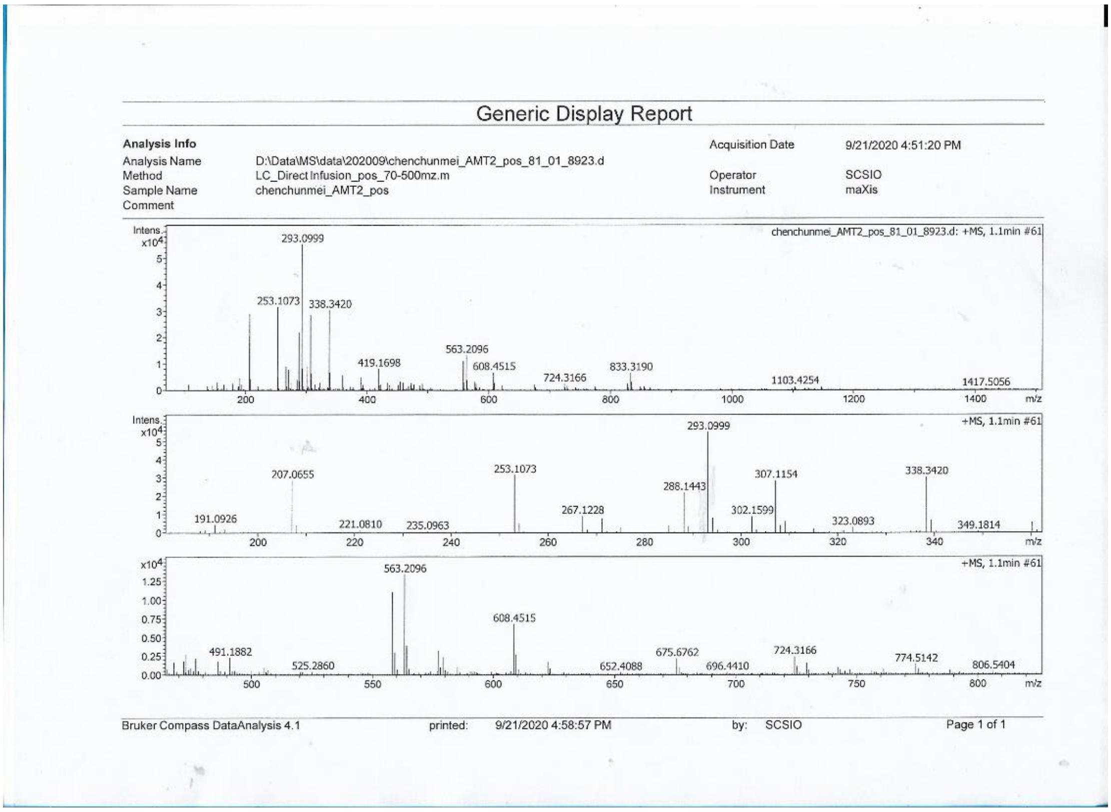

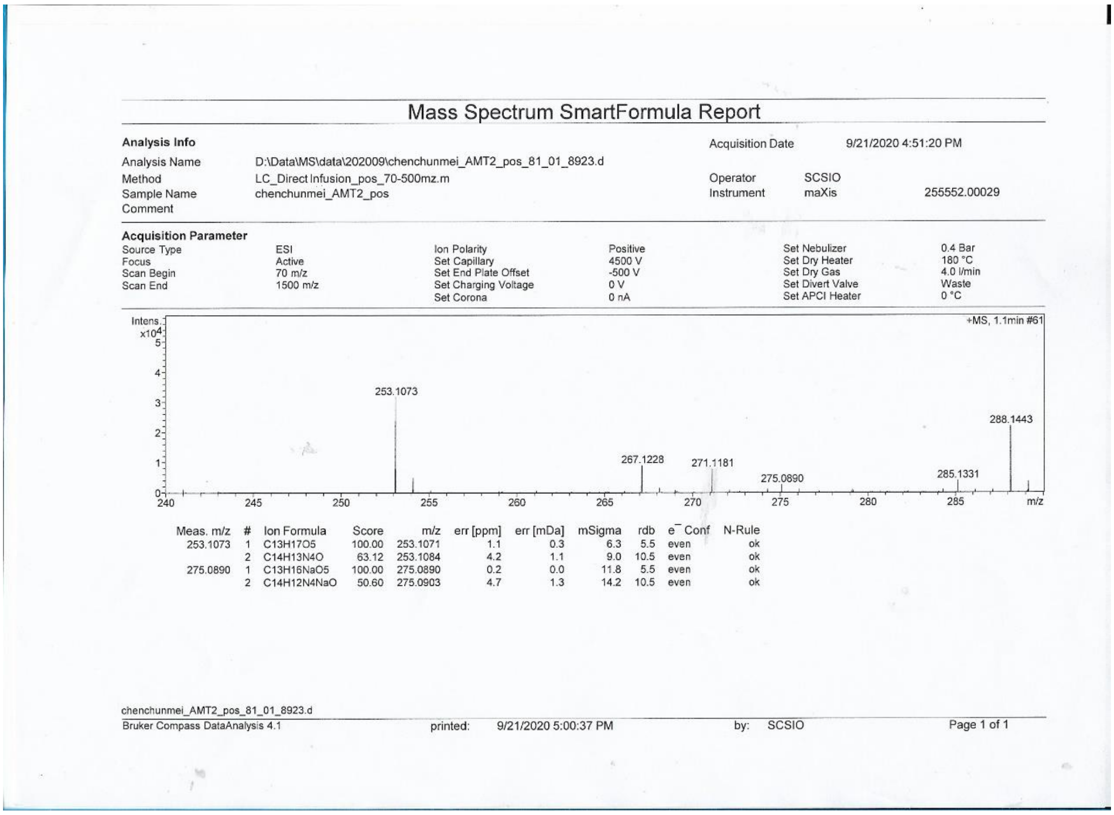

Figure S39. HRESIMS spectrum of maleicanhydridane (**4**).

ECD Calculation Details for 5.

The ECD spectra were generated based on Boltzmann distribution theory by the SpecDis 1.71 under a half band width of 0.18 eV and shifted by 0 nm to facilitate comparison to the experimental data.

Table S1. Energies of **5** at MMFF94 force field.

Configuration	Conformer	Energy (kcal/mol)	Population (%)
(7*R*,8*S*)-**5**	1	252.95	51.6
(7*R*,8*S*)-**5**	2	253.12	48.1
(7*R*,8*S*)-**5**	3	266.20	0.2
(7*S*,8*R*)-**5**	1	252.95	51.6
(7*S*,8*R*)-**5**	2	253.12	48.1
(7*S*,8*R*)-**5**	3	266.20	0.2

Table S2. Energies of **5** at B3LYP/6–31+g(d) level in methanol.

Configuration	Conformer	E (Hartree)	E (kcal/mol)	Population (%)
(7*R*,8*S*)-**5**	1	–840.8541634	–527644.396075134	49.99
(7*R*,8*S*)-**5**	2	–840.8541634	–527644.396075134	49.99
(7*R*,8*S*)-**5**	3	–840.8465769	–527639.635470519	0.02
(7*S*,8*R*)-**5**	1	–840.8541634	–527644.396075134	49.99
(7*S*,8*R*)-**5**	2	–840.8541634	–527644.396075134	49.99
(7*S*,8*R*)-**5**	3	–840.8465769	–527639.635470519	0.02

(7*R*,8*S*)-**5**

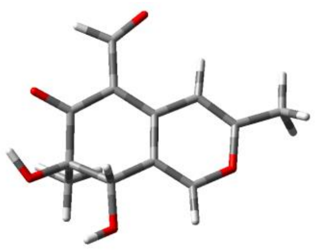	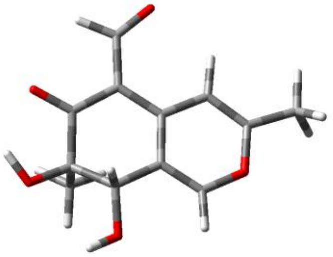	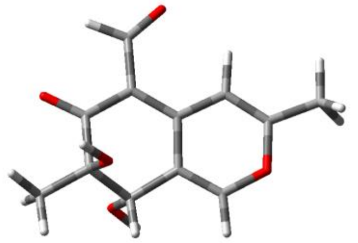
Conf.1 (49.99%)	Conf.2 (49.99%)	Conf.3 (0.02%)

(7*S*,8*R*)-**5**

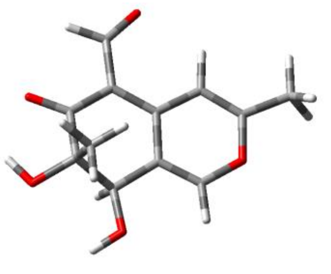	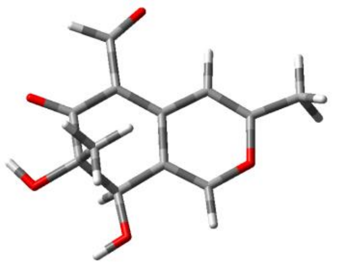	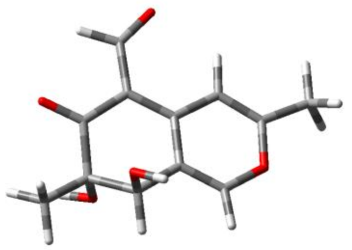
Conf.1 (49.99%)	Conf.2 (49.99%)	Conf.3 (0.02%)

Figure S9. The optimized conformers and equilibrium populations of **5**.

The strain's (*Talaromyces* sp. SCSIO 41050) ITS sequence of the rDNA

TACTTCCGTAAGGGGGACCTGCGGAAGGATCATTACCGAGTGAGGGCCCTC
GCGGCCCAACCTCCCACCCTTGTCTCATATACCTGTTGCTTCGGCGGGCCC
ACCGGGGCCACCTGGTCGCCGGGGGACGTCTGTCCCCGGGCCCGCGCCCG
CCGGAGCGCCCTTTGAACCCTGATGAAGATGGGCTGTCTGAGTGATATGAA
AATTGTCAAAACTTTCAACAACGAATCTCTTGGTTCCGGCATCGATGAAGA
ACGCAGCGAAATGCGATAAGTAATGTGAATTGCAGAATTCCGTGAATCATC
GAATCTTTGAACGCACATTGCGCCCCCTGGCATTCCGGGGGGCATGCCTGT
CCGAGCGTCATTTCTGCCCTCAAGCACGGCTTGTGTGTTGGGTGTGGTCCC
TCCGGGGACCTGCCCGAAAGGCAGCGGCGACGTCCGTCTGGTCCTCGAGC
GTATGGGGCTCTGTCACTCGCTCGGGAAGGACCTGCGGAGGTTGGTCACC
ACCACATCTTTTTACAAGGTTGACCTCGGATCAGGTAGGAGTTACCCGCT
GAACTTAAGCATATCTAGACGCCGGAGGAA

The strain's (*Penicillium* sp. SCSIO 41411) ITS sequence of the rDNA

TGATATGCTTAAGTTCAGCGGGTATCCCTACCTGATCCGAGGTCAACCTGAG
AAAGATTGAGGGGGGTCGCCGGCGGGCGCCGGCCGGGCCTACAGAGCGG
GTGACGAAGCCCCATACGCTCGAGGACCGGACGCGGTGCCGCCGCTGCCT
TTCGGGCCCGCCCCCCGGTAGCCGGGGGGCGGGGCCCAACACACAAGCCG
TGCTTGAGGGCAGCAATGACGCTCGGACAGGCATGCCCCCCGGAATACCA

GGGGGCGCAATGTGCGTTCAAAGACTCGATGATTCACTGAATTCTGCAATT

CACATTACTTATCGCATTTCGCTGCGTTCTTCATCGATGCCGGAACCAAGAG

ATCCGTTGTTGAAAGTTTTAACTGATTTAGCTAATCGCTCAGACTGCATTCT

TCAGACAGCGTTCAATGGTGTCTTCGGCGGGCGCGGGCCCGGGGGCGGAT

GCCCCCCGGCGGCCGGAACGGCGGGCCCGCCGAAGCAACAAGGTACGATA

AACACGGGTGGGAGGTTGGACCCAGAGGGCCCTCACTCGGAATGATCCTT

CCGCA
